# Colistin Resistant *A. baumannii*: Genomic and Transcriptomic Traits Acquired Under Colistin Therapy

**DOI:** 10.3389/fmicb.2018.03195

**Published:** 2019-01-07

**Authors:** Viviana Cafiso, Stefano Stracquadanio, Flavia Lo Verde, Giacoma Gabriele, Maria Lina Mezzatesta, Carla Caio, Giuseppe Pigola, Alfredo Ferro, Stefania Stefani

**Affiliations:** ^1^Department of Biomedical and Biotechnological Sciences, University of Catania, Catania, Italy; ^2^Department of Clinical and Experimental Medicine, University of Catania, Catania, Italy

**Keywords:** *A. baumannii*, colistin-resistance, genomics, transcriptomics, signatures

## Abstract

Even though colistin-based treatment represents the antimicrobial-regimen backbone for the management of multidrug-resistant Gram-negative infections, colistin resistance is still rare, at least as a full resistance, in *Acinetobacter baumannii* (*Ab*). We investigated the genomics and transcriptomics of two clinical Extensively Drug Resistance (XDR) colistin-susceptible/resistant (COL-S/R) *Ab* strain-pairs in which COL-resistance was developed after exposure to colistin therapy. The molecular characterization of the strains showed that all strains belonged to PFGE-A, ST-281, OXA-23 producers, Global Clone-II, and were resistant to imipenem, meropenem, ampicillin/sulbactam, ciprofloxacin, gentamicin, amikacin, trimethoprim/sulfamethoxazole, and susceptible to tigecycline, in agreement with NGS-acquired resistome. COL-R vs. COL-S *Ab* comparative genomics, mapping on *Ab* ATCC 17978 and *Ab* ACICU Reference Genomes, revealed a closely related genomic phylogeny, especially between strain-pair isolates, and distinctive common genomic non-synonymous SNPs (nsSNPs) in COL-R *Ab* strains. Furthermore, *pmrB* and *pmrC* nsSNPs were found. Notably we recovered, for the first time, *lpxC* and *lpxD* nsSNPs previously described only in “*in-vitro*” mutants and associated with colistin resistance in a clinical COL-R *Ab*. COL-R vs. COL-S *Ab* comparative transcriptomics evidenced a strain-dependent response to the colistin resistance onset highly variable among the single COL-R strains vs. their COL-S parents and merely seven common over-expressed transcripts, i.e. the PgaB lipoprotein for biofilm-matrix production, the diacylglycerol kinase for the lipid recycling in the membrane-derived oligosaccharide cycle, a membrane non-ribosomal peptide synthetase, the Lipid A phosphoethanol aminotransferase PmrC, and three hypothetical proteins. The transcript analysis of the “COL-R related genes” and the RNA-seq data confirmed *pmrCAB* over-expression responsible for a greater positive net cell-charge, and *lpxACD* under-expression in COL-R causing a decreased LPS production, as main mechanisms of colistin resistance. Our study reports the COL-R *Ab* genomic and transcriptomic signatures reflecting the interplay between several direct and indirect potential adaptations to antimicrobial pressure, including the occurrence of SNP accumulation hotspot loci in genes related to intrinsic or adaptive colistin resistance, surface adhesion proteins and porins, and over-expressed genes involved in different pathways, i.e. biofilm production, oxidative stress response, extensive drug and COL resistance.

## Introduction

The multi-drug resistant nosocomial pathogen *Acinetobacter baumannii* (*Ab*) represents an increasing global health threat. Colistin remains the last resort among single-agent therapies often combined with other antimicrobial agents (Vila and Pachón, 2012), even though colistin associated or antimicrobial combined therapies were clinically used as alternative antimicrobial regimens (Durante-Mangoni et al., 2014). This drug is a member of the polymyxin family of cationic polypeptides with a broad spectrum of activity against Gram-negative bacteria (Zavascki et al., 2007). Polymyxin antibacterial activity involves an initial interaction with the polyanionic lipopolysaccharide (LPS) within the outer membrane. Divalent cations that usually cross-bridge adjacent LPS molecules necessary for membrane stabilization are subsequently displaced (Hancock and Chapple, 1999) allowing the self-promoted uptake of polycationic peptides across its surface.

Although polymyxin resistance rates in surveillance studies remain very low (Gales et al., 2011), the increasing use of colistin coupled with the clonal spread of colistin-resistant strains could lead to an amplified trend that is already observed, for example in South Korea (27.9% of COL-R) (Ko et al., 2007).

Two main mechanisms of colistin resistance were previously described in *Ab* (Adams et al., 2009; Moffatt et al., 2010). Adams et al. (2009) demonstrated that resistant mutants could be generated under colistin pressure. These mutants contained mutations in the *pmrB* gene, with one mutant containing an additional *pmrA* mutation. Moffatt et al. (2010) suggested that the basis for polymyxin resistance in *Ab* was due to mutations in the first three genes (*lpxACD*) in the lipid A biosynthesis pathway. The study also demonstrated that the occurrence of these mutations led to the complete loss of LPS production and a greater susceptibility to other antibiotics. However, two additional, yet relatively poorly understood, resistance phenotypes, named hetero- and adaptive-resistance, have been reported in *Ab* (Falagas et al., 2010; Cai et al., 2012). Colistin hetero-resistance was first described by Li et al. (2006) and related to the emergence of a subpopulation from an otherwise susceptible (MIC ≤ 2 mg/L) population, the molecular mechanism involved in this phenomenon remains to be elucidated, and its understanding is critical due to the clinical significance of colistin hetero-resistance. Adaptive colistin resistance in *Ab* refers to a rapid induction of resistance in the presence of antibiotic and a reversal in its absence, moreover the adaptive resistance molecular mechanism involved in its onset remains to be elucidated (Olaitan et al., 2014).

An intrinsic colistin tolerance mechanism was also described and associated with more than 30 genes mainly associated with osmotolerance (Hood et al., 2013).

The transcriptome role in bacterial physiology and antimicrobial resistance mechanisms is becoming increasingly clearer for *Ab*. Some publications described the transcriptome contribution in the colistin resistance mechanism. Henry et al. (2012), working on *Ab* ATCC 19606 and its LPS-deficient *lpxA* mutant strain 19606R, demonstrated that in response to total LPS loss *Ab* alters the expression of critical transport and biosynthesis systems associated with modulating the composition and structure of the bacterial surface. Park et al. (2015), studying one COL-S and two COL-R *Ab* strains, found that the differentially expressed genes (DEGs) were all associated with either LPS biosynthesis or electrostatic changes in the bacterial cell membrane; LPS modification represents one of the principal modes of acquisition of colistin resistance in some *Ab* strains. Cheah et al. (2016) found that the transcriptomes of stable and non-stable polymyxin-resistant samples were not substantially different and featured an altered expression of genes associated with outer membrane structure and biogenesis. Using transcriptomic data, Wright et al. (2017) showed differential gene expression patterns related to mutations in the *pmrAB* and *adeRS* two-component regulatory system genes, as well as significant differences in genes related to antibiotic resistance, iron acquisition, amino acid metabolism, and surface-associated proteins.

In this study, using high-throughput-technologies such as NGS, RNA-seq and real time qPCR, two isogenic pairs of XDR COL-S and COL-R *Ab* clinical strains were investigated for genomic and transcriptomic characterization to gain new insights into the distinctive signatures of colistin resistant *Ab*.

Our data delineated the genomic and transcriptomic features of COL-R *Ab* strains, evidencing traits related to their complexity comprising different aspects of the *Ab* biology.

Our investigations focused different signatures of colistin resistant *Ab* consisting of common non-synonymous (ns) genome SNPs (gSNPs), especially in glutamate 5-kinase related to the intrinsic colistin resistance, *pmrBC* and *lpxC/D* along with their differential expressions, and expression changes in several genes implicated directly (ACICU_02907 diacylglycerol kinase and *pmrC*) or indirectly (A1S_2651 membrane non ribosomal peptide synthetase and ACICU_01518 hypothetical protein) in colistin resistance. Accessory traits evidencing signatures and pathways related to potential adaptation of this microorganism were also found.

## Materials and Methods

### Bacterial Strains, Growth Conditions

Two isogenic pairs of unrelated Italian COL-S/R clinical *Ab* strains (1-S/R, 2-S/R) were previously recovered from the bronchial aspirates of two patients hospitalized in two different Intensive Care Units (ICU) of a Sicilian hospital (Cannizzaro, Italy) being treated with colistin. The two isogenic pairs were not isolated by the authors but provided by Cannizzaro Hospital. Therefore, an ethics approval was not required as per institutional and national guidelines and regulations.

Isolates were collected by standard methods and identified at the species level using the API 20NE system (bioMérieux, Durham, NC, USA). Identification was also genomically confirmed by *de novo* whole genome sequencing data using the Speciesfinder tool (http://www.genomicepidemiology.org/). COL-S *Ab* ATCC 19606 was used as a control.

### Antimicrobial Susceptibility Tests

MIC assays for colistin (COL), imipenem (IMP), meropenem (MEM), ampicillin/sulbactam (SAM), ciprofloxacin (CIP), gentamicin (GEN), amikacin (AK), tigecycline (TGC), and trimethoprim/sulfamethoxazole (SXT) were performed using the standard broth microdilution method according to the European Committee on Antimicrobial Susceptibility Testing (EUCAST) and CLSI guidelines (Clinical and Laboratory Standards Institute. Performance standards for antimicrobial susceptibility testing; 20-third informational supplement. 2015; Document M100-S25).

TGC MIC determinations were performed using TGC purchased from Pfizer (Pfizer, Rome, Italy), whereas testing pertaining to COL, IMP, MEM, SAM, CIP, GEN, AK, and SXT was performed using powders purchased from Sigma (Sigma Chemical Co., St. Louis, MO, USA).

Susceptibility and resistance categories were assigned according to EUCAST breakpoints (http://www.eucast.org/clinical_breakpoints/). TGC breakpoints for *Enterobacteriaceae* (≤2/≥8 μg/mL for susceptible/resistant) established by the US Food and Drug Administration (FDA) were applied. *Escherichia coli* ATCC 25922 was used as the quality control strain.

### Whole Genome Sequencing (WGS)

Whole Genome Sequencing (WGS) was performed using the Illumina Mi-Seq sequencing system.

#### DNA Extraction

Genomic DNA was extracted from the samples using the GenElute Bacterial Genomic DNA kit (Sigma-Aldrich, UK), following the protocol for Gram negative bacteria. The quality of the DNA was verified using a 2200 TapeStation Genomic Screen Tape device (Agilent, Santa Clara, CA, USA) and its concentration ascertained by Picogreen (Life Technologies). DNA was normalized to 0.2 ng/μl in a volume of 50 μL. For paired end reads (PE), libraries were prepared by PGP with the Nextera XT DNA Library Prep Kit (Illumina, San Diego, USA) following the manufacturer's protocol and their evaluation was made with the Agilent Tape Station 2200. The indexed libraries were quantified with the ABI9700 qPCR instrument using the KAPA Library Quantification Kit in triplicate, according to the manufacturer's protocol (Kapa Biosystems, Woburn, MA, USA). Five μL of the pooled library at a final concentration of 2 nM were used for sequencing using Illumina Miseq with a 150 Paired-end Read sequencing module. For mate pair reads (MP), libraries were prepared by PGP with the Illumina Nextera Mate Pair Sample Prep Kit (Illumina, San Diego, CA, USA) following the manufacturer's protocol and their evaluation was made with the Agilent Tape Station 2200. The indexed libraries were quantified with the ABI9700 qPCR instrument using the KAPA Library Quantification Kit in triplicate, according to the manufacturer's protocol (Kapa Biosystems, Woburn, MA, USA). Five μL of the pooled library at a final concentration of 2 nM were used for sequencing using Illumina Miseq with a 300 Paired-end Read sequencing module.

#### Sample Preparation (*de novo* Sequencing)

The 4 samples were processed using the Illumina Mi-Seq technology, with two different libraries: (i) a paired-end library with reads of 150 bp and average insert size of 400 bp; (ii) a mate-pair library with reads of 250 bp and average insert size of 8 kb. After sequence data generation, raw reads were processed using FastQC v0.11.2 to assess data quality. The sequencing reads were then trimmed using Trimmomatic v.0.33.2 to remove only sequencing adapters for paired-end reads, while Mate Pair reads were processed by requiring a minimum base quality of 20 (Phred scale) and a minimum read length of 100 nucleotides, in order to filter out sequences composed only of Ns and to improve the per base score of the mate pair reads. Only trimmed reads were included in the downstream analysis. In S-Table_1, the total number of paired end and mate pair reads are reported with the estimated coverage.

#### *De novo* Genome Assembly

Genome assembly was performed using the SPAdes v3.5 software. Reads were initially normalized with khmer 1.3, and then error-corrected using the SPAdes Bayesian Hammer utility. Finally, the assembly was performed using recommended parameters for such Illumina data. The SPAdes software produced a contigs file for each sample. Post-assembly controls and metrics were generated using the Quast v.2.3 software. S-Table_2 shows the assembly statistics.

#### *De novo* Gene Annotation

The assembled scaffolds were processed using Prokka v1.9 software to predict genes and annotate those sequences using a set of conserved prokaryotic genes. Figures S1–S4 show the *de novo* Genome Assembly Report of the strains in study.

#### DNA-Sequencing Data Accession Number

The genomic sequence reads were deposited in the NCBI Genome database in the Sequence Read Archive (SRA) under study accession n° SRP133297.

#### Single Nucleotide Polymorphisms (SNPs)

For the SNP call, a genomic re-sequencing was performed from the paired-end library raw reads (acc. n° SRP133297). Illumina raw reads were trimmed using “Trimmomatic” requiring a minimum base quality of 20 (Phred scale) and a minimum read length of 36 nucleotides. Only trimmed reads were included in the downstream analysis. Each sample was aligned with the *Ab* ATCC 17978 reference genome (RefGen) sequence (CP000521.1), chosen because the most important mutations associated with resistance to colistin were annotated on this *Ab* genome (Arroyo et al., 2011; Traglia et al., 2014) and with *Ab* ACICU (CP000863.1), using “Bwa mem” ver 0.7.5a (Wright et al., 2017).

For detection of SNPs, and insertions and deletions (indels), each.bam file was sorted using Samtools v.0.1.19 and duplicated reads were marked using the Picard Mark Duplicates utility. Complex variants, SNPs and indels were detected using “Freebayes” v.0.9.14, which required a minimum mapping quality of 30 (Phred scale) and a minimum base quality of 20.

In S-Table_3, we report a coverage estimation for the reference genomes of the sample. Coverage is calculated using the formula: Cov = (N° mapped Reads × Reads Length/Reference Length), where the Read Length is the estimation of read size after the trimming step, and Reference Length is the appropriate reference genome size of the sample.

The majority of the sequenced reads were properly aligned with the corresponding reference genome. Even if 1-R seems to have less aligned reads compared to the other samples, the estimated coverage of 20X is sufficient for variation calling analysis. Variation calling output data (.vcf files) were provided as Supplementary Table Files (S-Table_4-1S on *Ab* ATCC 17978 for the 1-S *Ab*, S-Table_5-1R on *Ab* ATCC 17978 for 1-R *Ab*, S-Table_6-2S on *Ab* ATCC 17978 for the 2-S *Ab*, S-Table_7-2R on *Ab* ATCC 17978 for the 2-R *Ab*, and S-Table_8-1S on *Ab* ACICU for the 1-S *Ab*, S-Table_9-1R on *Ab* ACICU for 1-R *Ab*, S-Table_10-2S on *Ab* ACICU for the 2-S *Ab*, S-Table_11-2R on *Ab* ACICU for the 2-R *Ab*).

In order to select those SNPs that were only present in the COL-R strains, all SNPs were computationally re-filtered. All non-synonymous SNPs present in COL-R isolates were confirmed by Sanger sequencing.

#### Molecular and Genomic Epidemiology

*Ab* isolates were examined for their genetic relatedness using PFGE. This was facilitated following the extraction of genomic DNA and subsequent digestion with ApaI (Mezzatesta et al., 2012).

Whole Genome Sequencing raw data were analyzed with the ResFinder (v2.1) server (http://www.genomicepidemiology.org/), for the detection of the acquired antimicrobial resistance genes (Zankari et al., 2012) using a 98% threshold for nucleotide sequence identity and 60% for the minimum length coverage. MultiLocus Sequence Typing (MLST) was performed using the MLST (v1.8) server (http://www.genomicepidemiology.org/) with the Oxford University and Pasteur Institute (PI) scheme (Larsen et al., 2012).

#### Phylogenetic Trees

**Figure 1:**
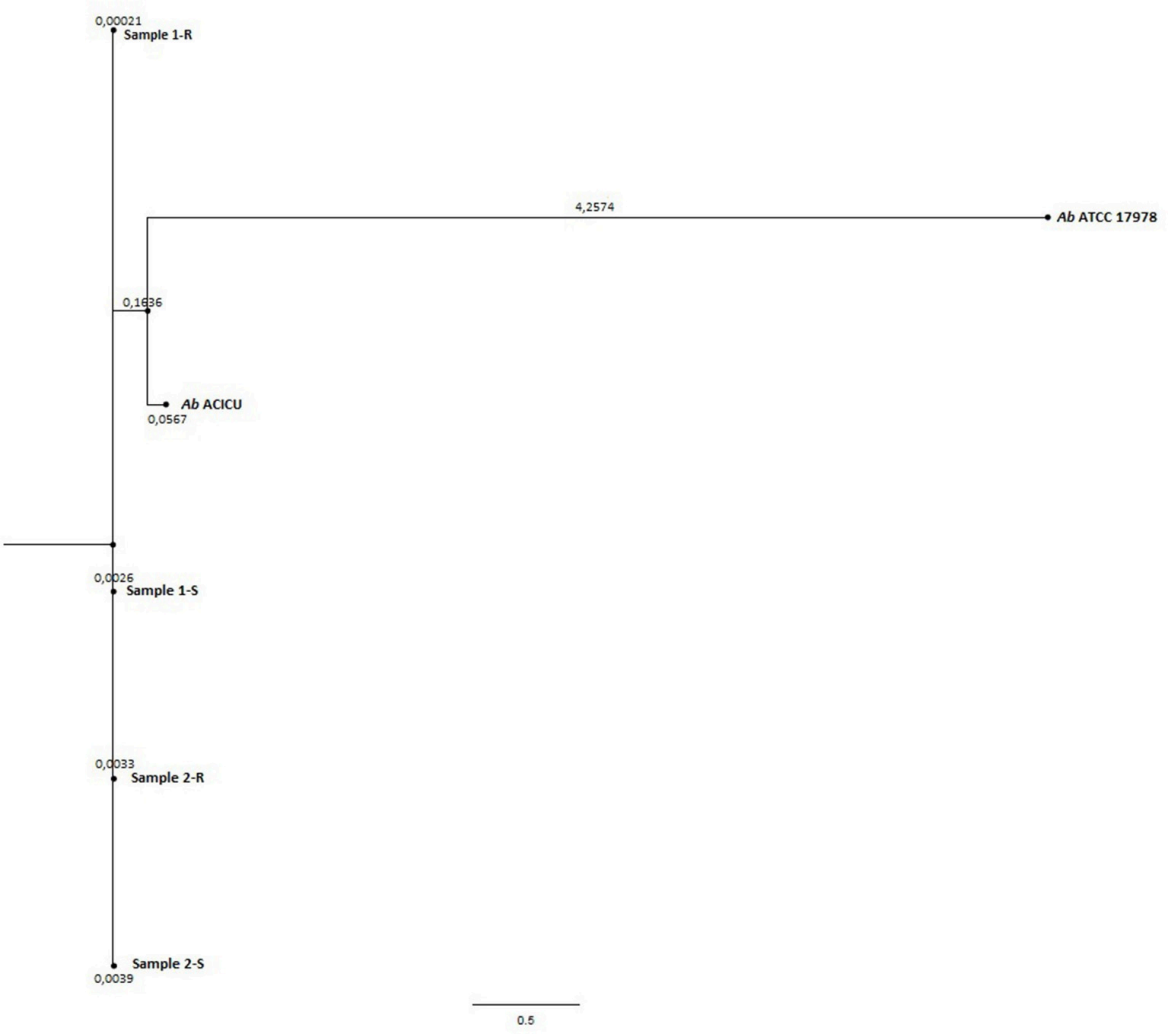
Phylogenetic tree (CSI-Phylogeny Tree).

**Figure 2:**
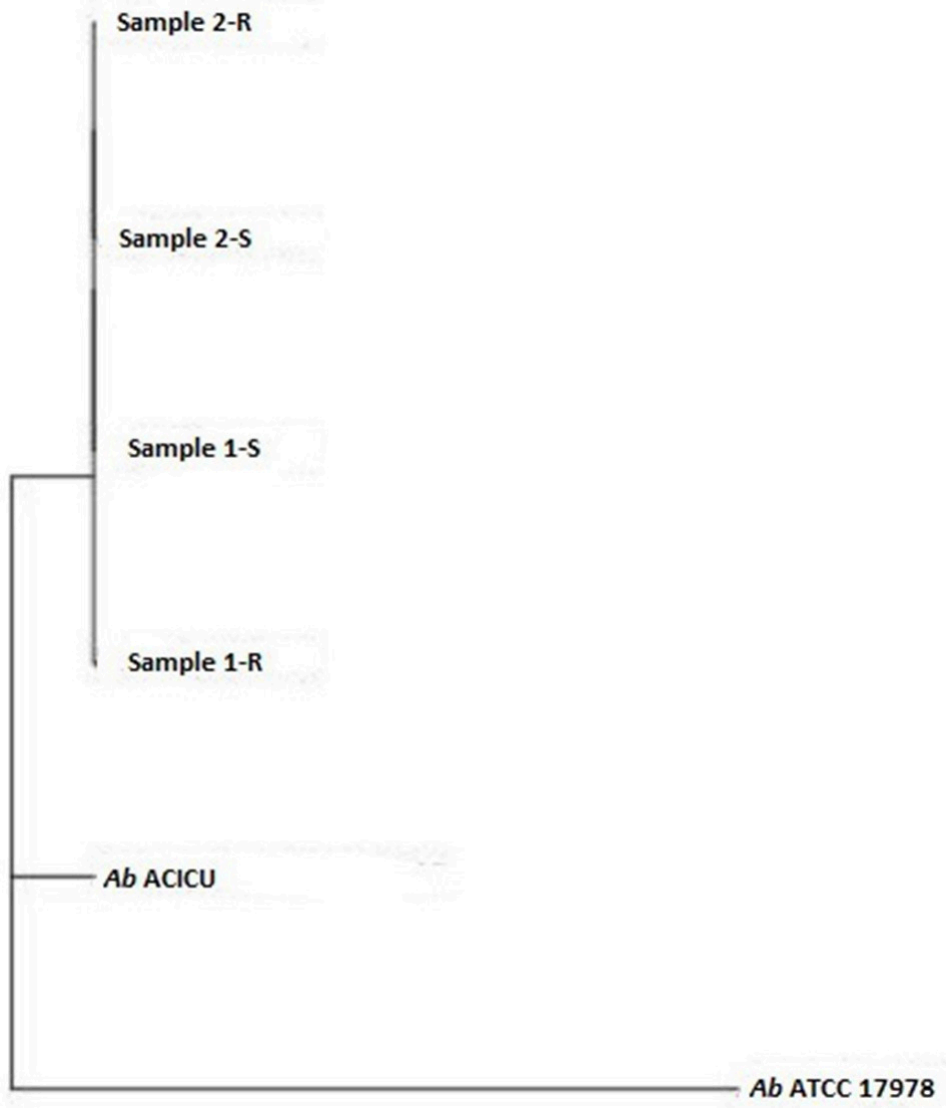
Phylogenetic tree (Realphy Tree).

Phylogeny was inferred by the whole genome sequencing raw data analysis with the CSI phylogeny on-line tool accessible on the Center for genomic epidemiology website (Kaas et al., 2014) and by the REALPHY pipeline, from the Swiss Institute of Bioinformatics, for a reference sequence alignment based Phylogeny builder that can infer phylogenetic trees from whole genome sequence data (Bertels et al., 2014). Both analyses were performed with default setting parameters. The graphical output of the two analysis was a Phylogenetic tree (Figures 1, 2).

#### Core SNPs

The core SNPs detection shared among all strains was computationally carried out by Nullarbor (http://github.com/tseemann/nullarbor).

### RNA-Seq

RNA-seq was performed using the Illumina Mi-seq sequencing system and two replicates were performed using two different libraries, a Single-End Library with 50 bp reads (Short-Insert Library) and a Paired-end Read Library with 150 bp reads (Tru-seq Library), as a strategy to optimize the collected RNA-seq data according to the following protocols.

#### Tru-Seq Library RNA Extraction

RNAs were extracted from the 4 samples grown until mid-log phase using the NucleoSpin RNA kit (Macherey-Nagel, Dueren, Germany) following the manufacturer's protocol with minor modifications. Bacterial cell pellets were lysed by the bead-beating procedure in the presence of 50 μL RA1 Buffer. Then 3.5 μL ß-mercaptoethanol were added and the lysate was filtered through NucleoSpin Filters (violet rings). RNA binding condition was adjusted by adding 350 μL of ethanol (70%) to the lysate and the RNA was then extracted following the protocol. The quality of the total RNA was verified using a 2200 TapeStation RNA Screen Tape device (Agilent, Santa Clara, CA, USA) and its concentration ascertained using an ND-1000 spectrophotometer (NanoDrop, Wilmington, DE, USA). Ribosomal RNA was removed using the Ribo-Zero rRNA Removal Kit (Bacteria) from 2 micrograms of total RNA, and the depleted RNA was used as input in the Illumina TruseqRNA stranded kit without PolyA-enrichment. The prepared libraries were evaluated with the High sensitivity D1000 screen Tape (Agilent Tape Station 2200). The indexed libraries were quantified with the ABI9700 qPCR instrument using the KAPA Library Quantification Kit in triplicate according to the manufacturer's protocol (Kapa Biosystems, Woburn, MA, USA). Five μL of the pooled library at a final concentration of 2 nM were used for sequencing using Illumina Miseq with a 150 Paired-end Read sequencing module.

#### Short-Insert Library RNA Extraction

RNAs were extracted from the 4 strains grown until mid-log growth phase and total RNA was extracted with Trizol reagent (Invitrogen) according to the manufacturer's protocol. After ribosomal depletion, sequencing libraries were prepared using the Illumina mRNA-seq sample preparation kit following the supplier's instructions except that total RNA was not fragmented and double-stranded cDNA was size-selected (100 to 400 bp) to also maximize the recovery of small-RNAs.

The prepared libraries were evaluated with the High sensitivity D1000 screen Tape (Agilent Tape Station 2200). The indexed libraries were quantified in triplicate with the ABI7900 qPCR instrument using the KAPA Library Quantification Kit, according to the manufacturer's protocol (Kapa Biosystems, Woburn, MA, USA). From the pooled library, 5 μL at a final concentration of 4 nM were used for Miseq sequencing with an A single end stranded library with reads of 50bp sequencing module.

#### Tru-Seq Library Preparation and Sequencing

The samples were processed using the Illumina MiSeq technology, using an A paired-end library with reads of 150 bp and average insert size of 350/400 bp. After sequence data generation, raw reads were processed using FastQC v0.11.2 to assess data quality. The sequenced reads were then trimmed using Trimmomatic v.0.33.2 to remove only sequencing adapters for Paired-end reads.

A minimum base quality of 15 over a 4-bases sliding-window was required. Only sequences with a length above 36 nucleotides were analyzed. Only trimmed reads were included in the downstream analysis.

#### Short-Insert Library Preparation and Sequencing

The samples were processed using the Illumina MiSeq technology with an A single end stranded library with reads of 50 bp. After sequence data generation, raw reads were processed using FastQC v0.11.2 to assess data quality. Reads were then trimmed using Trimmomatic v.0.33.2 to remove sequencing adapters for Single-end reads, requiring a minimum base quality of 15 (Phred scale) and a minimum read length of 15 nucleotides. Only trimmed reads were included in the downstream analysis.

#### Tru-Seq and Short-Insert Library Analysis

Tru-seq and Short-insert RNA-seq reads were aligned on *Ab* ATCC 17978 (CP000521.1) and on *Ab* ACICU (CP000863.1) RefGen, as well as transcripts assembled and quantified using Rockhopper v.2.03 (McClure et al., 2013; Tjaden, 2015) specifically designed for the bacterial gene structures and transcriptomes. Analyses were run on default parameter settings with verbose output to obtain expression data. Rockhopper normalizes read counts for each sample using the upper quartile gene expression level. Starting from the *p*-values calculated according to the Anders and Huber approach, DEGs were assigned by computing *q*-values ≤ 0.01 based on the Benjamini–Hochberg correction with a false discovery rate of <1%. In addition, Rockhopper is a versatile tool using biological replicates when available, and surrogate replicates when biological replicates for two different conditions are unavailable, considering the two conditions under investigation surrogate replicates for each other (McClure et al., 2013).

Finally, filtering analyses were computationally carried out for sorting, first, the differentially expressed genes in the COL-R strains vs. their COL-S parental strains and, then, the selection of only those present contemporarily in both COL-R strains showing the same expression (up- or down-regulation).

#### Determination of RNA Functional Categories

Functional categories of the genes with differential expression in COL-R vs. COL-S parents were investigated by bioinformatics including BLAST, PANTHER (Protein ANalysis THrough Evolutionary Relationships) Classification System, Gene Ontology (GO) Consortium, ExPASy, STRING and Kyoto Encyclopedia of Genes and Genomes (KEGG).

#### Determination of the Affected Pathways

Online tool DAVID (http://david.abcc.ncifcrf.gov/) (Huang et al., 2009a,b) was selected to identify the affected pathways among the DEGs. The gene lists were uploaded as Official Gene Symbols of the reference genome *Ab* ATCC 17978 or *Ab* ACICU, and automatically selecting the list type (Gene list) of *A. baumannii*. Functional Annotation Chart was visualized using the *p*-value threshold of 0.01 and a minimum count number of 4 genes. The information on the affected pathways was obtained from KEGG within the analysis in DAVID, using the mentioned thresholds. The affected biological processes were obtained from Gene Ontology Consortium within the analysis in DAVID.

#### RNA-Sequencing Data Accession Number

RNA-seq data of the two libraries were deposited in the NCBI Gene Expression Omnibus (GEO) database under the accession number GSE109951.

#### I-Tasser *ab initio* Structure Modeling

For the I-Tasser (Iterative Threading asseMBLY Refinement) analysis (Roy et al., 2010) the MKLKTVTIDGKVYAEVDGDKPIYIHDDGKEMPHDAPHS- VATIARLNNEAKTHREAKEAAKALKAFEGIEDPAAAKKALQTIQNLDDKKLVDAGEVEKVKAEAIKAVEEKYAPIVAQRDALEASLHKELIGGGFARSKYIQDNIAVPVDMVQATFGHHFKIEEGKVVAYDPNGEKIYSRVRPGELANVDEALESLVGGYQHKDLILKGGKGTGGGFQGGGKGGAPTGMKRSEMSVSQKADYIKEHGNDAFLKLPN A1S_2027 was selected as the target, where as the MNTLNINDIKKHADVIASCGTKVGTVDHLEGENQLKLTK- DENDQHHLIPTSWIGEVKEDVILNKNSEEVKENWQAI for A1S_2230, and MKKLSTILTAGVLAMLSVSAFACPKGTQLQGGTGPNHKGGKCVAVHGKATAQKAKKEATKTKQEVKKDLTMQKHDAMTSATHAQHESHQMTHQMKQDAVKTANTAKAATKP for ACICU_01518.

### Real Time qPCR

The expression of the A1S_0938, A1S_2027, A1S_2230/ACICU_02436, A1S_2651, A1S_2752/ACICU_03004 genes showing differences in both COL-R strains compared with the COL-S strains after RNA-seq, as well as *lpxACD* and *pmr*-operon transcript quantification were validated and analyzed by real-time qPCR.

Overnight cultures of all strains were diluted 1:50 in Cation-adjusted Mueller–Hinton broth (Ca-MHB) and grown until the mid-log phase (OD_600_ = 0.5) for RNA-seq data validation, and until the exponential phase (OD_600_ = 0.04-0.07) and mid-log phase (OD_600_ = 0.5) for the investigations on *lpxACD* and *pmr*-operon transcription. RNA was subsequently extracted, treated and quantified as previously described (Cafiso et al., 2012, 2014). mRNA was retro-transcribed and real-time qPCR was carried out using a previously published method (Cafiso et al., 2012, 2014).

All real time qPCRs were performed in triplicate using an initial denaturation stage of 95°C for 3 min, followed by 35 cycles at 95°C for 10 s, 60°C for 30 s, 72°C for 45 s and a final cycle at 95°C for 1 min, and from 60°C for 30 s to 95°C for 30 s. The primers used to generate products for quantification resulted in the amplification of a fragment of less than 260 bp. *rpoB* was used as a normalizer and constituted the internal control. All of the primers that were used for the real time qPCR study are listed in S-Table_12. For each analysis, three to five distinct biological replicates were performed. Statistical expression analyses were conducted with REST2009 tool as previously described (Pfaffl et al., 2002).

The real time qPCR expression levels of *lpxACD* and the *pmr*-operon were represented as the increment/decrement fold-change (FC) in COL-R (1-R, 2-R) vs. COL-S isolates (1-S, 2-S). Expression level analysis was performed for the exponential and mid-log growth phase cultures.

The real time qPCR expression level of the A1S_0938, A1S_2027, A1S_2230/ACICU_02436, A1S_2651, A1S_2752/ACICU_03004 genes, for the RNA-seq data validation, were shown as FCs of COL-R strains vs. the COL-S strains in RNA-seq vs. real time qPCR. Three biological replicates were considered.

### Surface Charge Determination

The cytochrome *c* binding assay was performed as a surrogate measure of the relative net positive surface charge of the strain-pairs using a previously described procedure (Yang et al., 2009, 2010, 2012). The amount of unbound cytochrome *c* that was detected in the supernatant correlated directly with the net positive charge associated with the bacterial surface. The generated data are expressed as the mean amount of bound cytochrome *c* ± SD. A minimum of three independent experimental repeats were performed on at least three separate days.

### LPS Amount and Surface-Attached Polysaccharides Quantification

Lipopolysaccharide (LPS) was extracted by the hot phenol-water method as previously described (Rezania et al., 2011). Extra pure *E. coli* LPS (Sigma) was used to prepare a standard curve to quantify extracted LPSs. For all samples and standards, OD_259_ was measured in a spectrophotometer (Thermo Scientific™ GENESYS 10S UV-Vis), and the standard curve used to determine LPS concentration in unknown samples. Surface-attached Polysaccharides (SP) extraction and quantification were carried out as described by Brimacombe and Beatty (2013).

LPS and SP extractions were performed during bacterial stationary growth phase and the cultures were normalized to OD_650_ 2.0 (Brimacombe and Beatty, 2013). Data are derived from at least three independent experiments for both methods.

## Results

The four *Ab* strains investigated were isolated from serial bronchial aspirates of two hospitalized patients. Patient 1 underwent a pulmonary lobectomy caused by a respiratory distress, whilst Patient 2 was a severely burned patient that had a cardiac arrest. Both patients were initially infected with COL-S *Ab*, consequently treated with colistin, and then infected with the COL-R *Ab*. The interval between the isolation of the COL-S and the COL-R *Ab* strain was 24 days for Patient 1 and 7 days for Patient 2. No reversion of colistin resistance upon colistin withdrawal was observed in both patients.

### Molecular Characterization-Genomic Epidemiology-Phylogeny

All isolates were designated ST-281 according to the Oxford University scheme and ST-2 according to the Pasteur Institute grouping, even though COL-R *Ab* 1-R was grouped as ST-187 with PI - a ST-2 Single Locus Variant harboring a nucleotide change resulting in the conversion of the *rpoB*_2 allele to the rpo*B*_43 allele. All COL-R *Ab* strains assigned to the same PFGE macrorestriction profile A and to the Global Clonal lineage II (Table 1).

**Table 1:** Molecular characterization, antibiotype and resistome of the *A. baumannii* strains.

**Strain**	**OU** ** MLST**	**PIMLST**	**PFGE**	**GC**	**MICs (mg/L)**									**Resistome**		
					**COL**	**IPM**	**MEM**	**SAM**	**CIP**	**GEN**	**AK**	**TGC**	**SXT**	**β-lactams**	**AGs**	**SAs**
1-S	281	2	A	II	0.125	16^*^	16^*^	>256^*^	>32^*^	8^*^	128^*^	2	8^*^	*blaADC-*25 *blaOXA-*23 *blaOXA-*82	*aadA2* *ant(2″)-Ia* *aph(3′)-Vla*	*sul1*
1-R	281	187	A	II	64^*^	16^*^	16^*^	>256^*^	>32^*^	16^*^	128^*^	2	8^*^	*blaADC-*25 *blaOXA-*23 *blaOXA-*82	*aadA2* *ant(2″)-Ia* *aph(3′)-Vla*	*sul1*
2-S	281	2	A	II	0.125	16^*^	16^*^	>256^*^	>32^*^	8^*^	64^*^	2	16^*^	*bla*ADC-25 *bla*OXA-23 *bla*OXA-82	*aad*A1 *aad*A2 *aac*(3)-Ia *ant(2″)-Ia* *aph*(3′)-VIa	*sul*1
2-R	281	2	A	II	256^*^	16^*^	16^*^	>256^*^	>32^*^	8^*^	32^*^	2	16^*^	*bla*ADC-25 *bla*OXA-23 *bla*OXA-82	*aad*A1 *aad*A2 *aac*(3)-Ia *ant(2″)-Ia* *aph*(3′)-VIa	*sul*1

* *indicates that the MIC value falls above the resistance breakpoints. OU, Oxford University; PI, Pasteur Institute; AGs, Aminoglycosides; SAs, Sulfonamides*.

**Figure 3:**
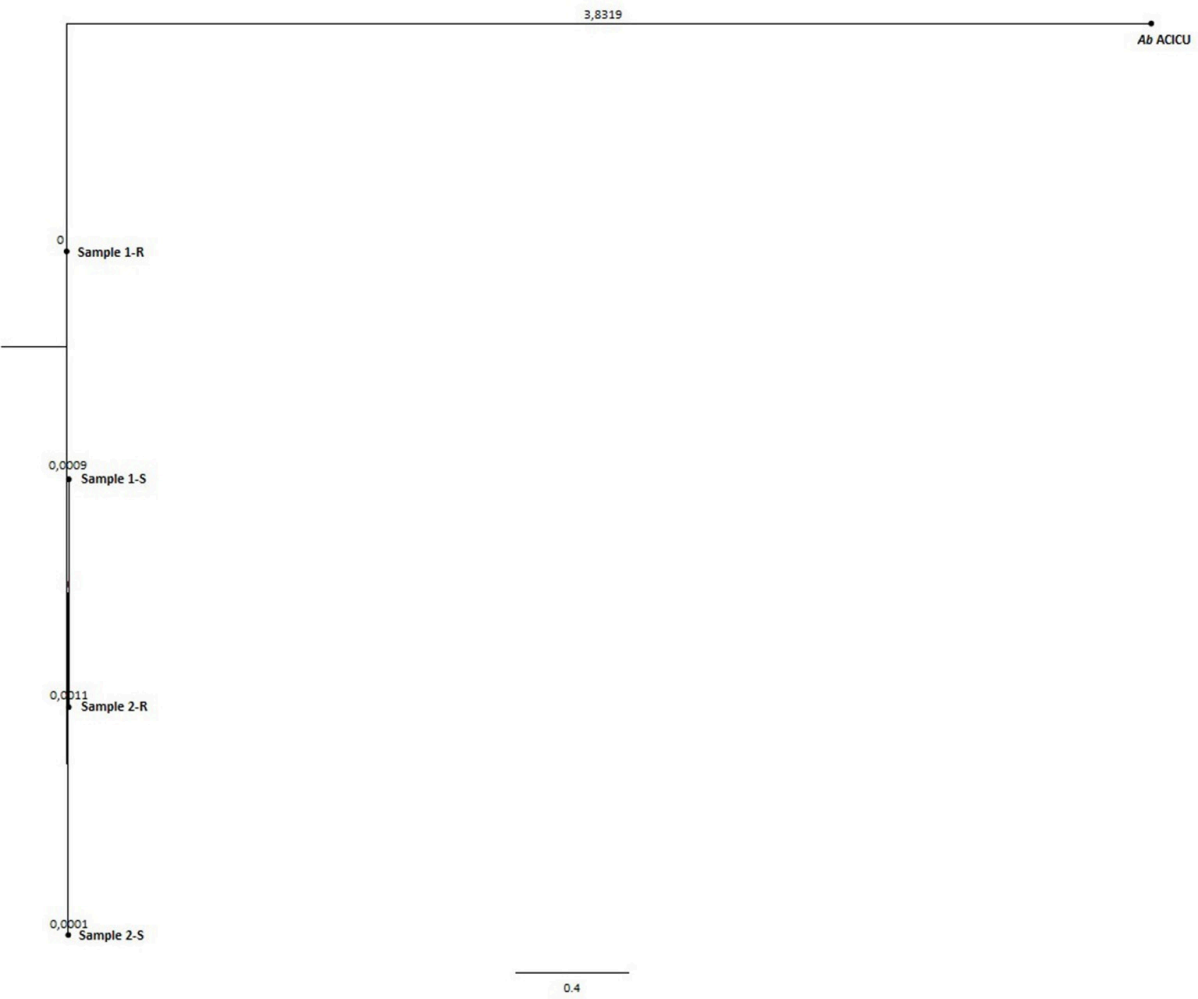
Phylogenetic tree (Core SNP Tree).

To deeply examine and clarify the phylogenetic relatedness among the COL-R and COL-S *Ab* strains recovered from the same patients, and their relation to Ref strains, we generated two whole gSNP phylogenetic trees by two different computational approach, i.e. a phylogeny based on the reference-based concatenated alignment of the high quality SNPs and a construction of a maximum likelihood tree from the alignment by using a modified, more accurate version of FastTree (CSI-PHYLOGENY) and a phylogeny reconstructed by combined alignments obtained by mapping reads to not one but to multiple reference sequences (REALPHY), shown in Figures 1, 2. Both gSNP phylogenetic trees showed a closely phylogeny of the COL-S and COL-R isolates belonged to the same strain pair along with a stronger correlation of two strain pairs with the *Ab* ACICU genome than the *Ab* ATCC 17978. The phylogenetic tree generated on core SNPs (cSNPs) confirmed and corroborated these considerations (Figure 3). Furthermore, the Nullarbor computational analysis, generating a core genome alignment and provided high quality core SNPs, evidenced an average of 11,236 cSNPs across 3,904,116 bp of the *Ab* ACICU RefGen shared among the 4 strains. Comparing pairwise cSNP differences and according to the criteria considering 1-40 cSNP difference range as indicator of a very high phylogenetic relation and 4-44830 cSNP difference range for genetically distinct phylogenetic subclades recently used to infer the phylogeny in colistin-resistant *Ab* (Mustapha et al., 2018), we considered the strains of the 1 pair as highly related strains, having 70 cSNP differences between the COL-R and COL-S parent strains, whereas the strains of the 2 pair as very highly related showing 18 cSNP differences. Furthermore, we found 23 cSNP differences comparing pairwise 1-S vs. 2-S, and 81 cSNP differences among the 1-R vs. 2-R (Table 2).

**Table 2:** AA changes emerging under therapy in COL-R *A. baumannii*.

**Strain**	**Core SNP difference**	**PmrB domains** ** and AA changes**				**PmrC domains** ** and AA changes**		**LpxC domains** **and AA changes**		**LpxD domains** **and AA changes**		**AA changes in** ** protein related** ** to colistin resistance** ** in COL-R** ** strains vs. COL-S** ** parental strain**
		(1-215)	HisK^C^ (216-276)	(277-330)	HTAPaseC^C^ (331-419)	(1-236)	Sulfatase^C^ (237-532)	PET N-terminal (44-194)	LptA (220-515)	UDP-3-O-(3-hydroxymyristoyl) Glucosamine N-acyltransferase (2-341) containing:		
										NAT non-repeat region (26-92)	Hexapeptide repeats (112-144) (148-183) (225-260) (262-288)	
1-R vs. 1-S	70	L208F (Arroyo et al., 2011) (This study)					L340F (This study)	S171Y (This study)		S292^*^ (This study)		- ProB:Y43S-V44I-E66Q (This study) - A1S_2928:F158Y,T163S, N166I (This study) - ACICU_02735: I120L (This study)
2-R vs. 2-S	18		R263H (Lim et al., 2015) (Choi et al., 2017) This study									

c = Previously predicted domains according to NCBI domain predictor (www.ncbi.nlm.nhi.gov/protein) are indicated as follows: Sulfatase domain; HisK, histidine kinase (dimerization/phosphoacceptor) domain; HTAPaseC, histidine-kinase-like ATPase. PET, Phosphoethanolamine transferase N-terminal; LptA, Lipooligosaccharide Phosphoethanolamine Transferase A or Lipid A Phosphoethanolamine Transferase catalyzes the modification of the lipid A headgroups by phosphoethanolamine (PEA) or 4-amino-arabinose residues (Interpro: https://www.ebi.ac.uk/interpro/). NAT = UDP-3-O-[3-hydroxymyristoyl] glucosamine N-acyltransferase non-repeat region. The amino acids (aa) corresponding to these domains are displayed in brackets.

* *Stop Codon*.

### Antibiotype

All strains included in this study were deemed XDR following comparison with previously agreed definitions (Magiorakos et al., 2012). MICs showed an uniform resistance profile to all antimicrobials, with the exception of TGC (MIC of 2 mg/L). Variations in this profile were only observed in the colistin susceptibility within the isogenic strains in both pairs (1-S and 2-S) (Table 1).

### Resistome

Resistome analysis refined the occurrence of the several acquired resistance genes including beta-lactam resistance ones, i.e. *blaADC*-25, *blaOXA*-82, and *blaOXA*-23 harbored in Tn2006 and flanked by two copies of the insertion sequence ISAba1 with an opposite orientation as in Refseq GQ861439.1, and the sulphonamide resistance gene *sul*1, in both *Ab* strain pairs. Different aminoglycoside resistance genes were also detected, with *aadA*2, *aph*(3′)-Vla and *ant*(2″)-Ia recorded in the 1-S/R *Ab* and *aad*A1, *aadA*2, *ant*(2″)-Ia, *aac*(3)-Ia, and *aph*(3′)-VIa in the 2-S/R *Ab* (Table 1). Regarding to Fluoroquinolone (FQ) resistance, no GyrA (ACICU_02869) AA changes were detected, whilst S84L in ParC (ACICU_00214) was found in all strains when annotated on *Ab* ACICU RefGen. All strains harbored T669S in AdeB (ACICU_01824), a resistance-nodulation-cell-division (RND)-type efflux pump related to resistance toward various antibiotics including FQs. Neither the “*qnr*-family” genes, *aac*(6′) Ib-cr, *norA, oqxA-B*, nor the *qepA-A*2 FQ-resistance genes were observed in *Ab* strains.

### Characterizing COL-R *Ab* gSNP Signatures

For the genomic SNP call, we preferred to assembly the sequencing raw data on two different reference genomes, i.e. *A. baumannii* ATCC 17978 and ACICU, since on *Ab* ATCC 17978 genome were firstly annotated the most important mutations associated with colistin resistance (Arroyo et al., 2011; Traglia et al., 2014), whilst the second genome was previously categorized as MLST Pasteur ST2, Oxford ST281, international clonal lineage II, as the *Ab* sample in study were categorized. Furthermore, *Ab* ACICU was used as RefGen in previous investigations on colistin-resistance in *Ab* (Wright et al., 2016).

In addition, a filtering of the gSNPs present only in both COL-R strains showed distinctive common genomic SNPs in COL-R *Ab* mapped both on RefGen *Ab* ATCC 17978 and RefGen *Ab* ACICU.

In details, the alignment on RefGen *Ab* ATCC 17978 displayed a total of 22 common synonymous and non-synonymous gSNPs in 10 genes and in three non-coding regions as shown in Table 3A. Among these, genomic re-sequencing data revealed six common nsSNPs conferring S316A change in FilD porin (long-chain fatty acid transport); E66Q in the A1S_2024 - homologous to the 5-glutamate kinase encoding gene of the *E. coli proB*- involved in intrinsic colistin resistance; L576F and N577D in the A1S_2443 surface adhesion protein; F158Y, T163S, and N166I in A1S_2928 hypothetical protein. Finally, three SNPs fell into three intergenic regions, with one located in the non-coding region predicted as the A1S_1091 succinylornithine transaminase promoter (carbon starvation protein C) (Table 3A).

**Table 3:** COL-R *Ab* common genomic SNPs.

***Ab* ATCC 17978 ** ** protein tag**	**SNP/indel ** ** genomic ** ** localization (nt)**	**SNP/indel**	**SNP function class ** ** (AA substitution)**	**Protein**
**(A)**				
**A1S_0693**	825375	TCCGCTAAA → GCAGCCAAG	***S316A***	**FilD**
	825389	AT → TC	sSNP	
	825405	T → A	sSNP	
Non-coding region	868391	T → C	–	–
A1S_0910	1054569	A → T	sSNP	Gamma-glutamyltranspeptidase
Non-coding region Promoter (-10) of A1S_1091	1273501	CGAG → TGAC	-	Succinylornithine transaminase (carbon starvation protein C)
A1S_1209	1414952	A → G	sSNP	Benzoate transport porin (benp)
	1414967	G → A	sSNP	
	1414979	CTTA → TTTG	sSNP	
A1S_1546	1800398	A → G	sSNP	Organic solvent tolerance protein
Non-coding region	1961637	TA → CT	–	–
**A1S_2024**	2351886	GC → GT	sSNP	**Glutamate 5-kinase**
	2351896	CGTCTCA → CGTCTGA	**E66Q**	
A1S_2029	2357676	AACG → CACA	sSNP	Hypothetical protein A1s_2029
	2357697	T → G	sSNP	
**A1S_2443**	2826634	TAAG → CAAA	**L576F/N577D**	**Surface adhesion protein**
A1S_2856	3304823	TAATTTGC → CAACCTGT	sSNP	Esterase
**A1S_2928**	3391139	T → A	**F158Y**	**Hypothetical Protein A1S_2928**
	3391149	T → A	**T163S**	
	3391154	C → G	sSNP	
	3391163	A → T	**N166I**	
A1S_3127	3612685	C → A	sSNP	Signal Peptide
***Ab*** **ACICU** **ProteinTag**	**SNP/indel** **genomic** **localization (nt)**	**SNP/indel**	**SNP function class** **(AA substitution)**	**Protein**
**(B)**				
**ACICU_01006**	1119168	CACGCT → AACACC	**S54G**	**Hypothetical protein**
**ACICU_01043**	1140525	ATGTCGAA → CTATTGAG	**Y43S/V44I**	**Glutamate 5-kinase**
**ACICU_01052**	1144802	GCA → ACT	**A65T**	**Hypothetical protein**
**ACICU_01060**	1152378	A → T	**I969L**	**Phage-related minor tail Protein**
ACICU_01554	1666862	A → C	sSNP	Biotin synthase
ACICU_01949	2080866	A → C	sSNP	Aldehyde dehydrogenase
ACICU_01985	2121627	T → G	sSNP	Iclr family transcriptional regulator
ACICU_02403	2540136	A → C	sSNP	Conserved uncharacterized protein
**ACICU_02735**	2908460	G → A	sSNP	**Hypothetical protein**
	2908471	TTAAC → GTAAT	**I120L**	
**ACICU_02736**	2909388	TACAGCA → AACTACG	**A153**	**Bacterial surface proteinContaining Ig-like domains**
**ACICU_02910**	3084936	A → G	sSNP	**Putative surface adhesion protein**
	3084942	T → A	sSNP	
	3084951	AGTG → GGTA	sSNP	
	3085085	A → C	**S245A**	
ACICU_02939 Intergenic region	3123710	A → G	-	-
ACICU_02943	3125312	A → G	sSNP	Hypothetical protein
ACICU_tRNA47	3391221	A → T	-	tRNA-ALA
	3391345	C → T	-	tRNA-ALA
ACICU_03509	3723733	A → C	sSNP	3-Deoxy-D-manno-octulosonicacid transferase
**ACICU_RS19025 (pACICU1)**	24584	T → G	**E92A**	**Hypothetical protein**
**ACICU_p0078 (pACICU2)**	45280	AT → A	**Y53T**	**Type IV secretion system** **Protein traC**

*sSNP, Synonymous SNP. The nsSNPs are reported in bold*.

SNP mapping on *Ab* ACICU RefGen showed also 22 different common synonymous and nsSNPs in 13 genes. nsSNPs were found in the 5-glutamate kinase, similarly to the *Ab* ATCC 17978 annotation, determining the Y43S and the V44I AA changes; A153V in ACICU_02736 bacterial surface protein containing Ig-like domains; S54G in ACICU_1006, A65T in ACICU_01052, I120L in ACICU_02735 hypothetical proteins; I969L in the ACICU_01060 phage related minor tail protein; S245A in ACICU_02910 putative surface adhesion protein (Table 3B).

Furthermore, one SNP were found in the ACICU_02938-ACICU_02939 intergenic region, two SNPs in the tRNA-Ala gene, one nsSNPs in pACICU1 and one in pACICU2, as shown in Table 3B. The pACICU1 nsSNP - causing an E92A AA change in the ACICU_RS19025 hypothetical protein homologous to YafO, type II toxin-antitoxin system region of other *Ab* plasmids. The pACICU2 nsSNP determining the Y53T change in Type IV secretion system protein TraC due to a deletion AT → T resulting in a frameshift causing a premature stop codon at the 58th aminoacid residue in both COL-R strains vs. the COL-S parent strains (Table 3B).

### SNPs of the Known Genes Associated With Colistin Resistance

In COL-R *Ab* vs. their COL-S parent strains mapped both on *Ab* ATCC 17978 and *Ab* ACICU RefGens, WGS data showed *pmrB* nsSNPs on *Ab* ATCC 17978 and *Ab* ACICU as follows: (i) L208F AA substitution in a domain without specific functions, in 1-R; (ii) R263H AA substitution in the PmrB histidine kinase domain, in 2-R.

In contrast, *pmrC* SNPs were only found on *Ab* ACICU annotation leading to L340F AA change in the PmrC sulfatase domain, coupled to a synonymous T → C SNP in 1-R, while a synonymous C → T was found in 2-R.

No *lpxACD* nsSNPs were observed in COL-R vs. COL-S strains with respect to the *Ab* ATCC 17978 RefGen, contrarily *lpx* cluster-nsSNPs were found on *Ab* ACICU RefGen in 1-R, determining the S171Y AA substitution in the LpxC phosphoethanolamine transferase N-terminal domain, and G → T in *lpxD* (ACICU_02090) leading to a truncated protein at the 292 AA (292 AA instead of 356 AA in *Ab* ACICU RefGen), due to a premature termination codon. No *lpxA* nsSNPs were found on *Ab* ACICU RefGen in 1-R and 2-R *Ab* strains (Table 2).

### Comparative Transcriptomics

To define the transcriptomic signatures of the two COL-R vs. COL-S *Ab* clinical strains, DEGs were determined from the RNomes by filtering firstly the differentially expressed RNAs in the COL-R strains vs. their COL-S parents and, then, re-filtering sorting those present contemporarily in both COL-R isolates with the same expression trend (over or under-expression).

From the Tru-seq (TS) Libraries, Illumina RNA-sequencing generated 1,307,792 - 1,175,327 - 1,173,332 - 1,270,020 total reads in 1-S, 1-R, 2-S, and 2-R, respectively, with 96, 96, 72, and 93% mapped reads on *Ab* ATCC 17978, as well as 97, 97, 76, and 95% reads aligned on *Ab* ACICU. RNome analysis on *Ab* ATCC 17978 revealed 47 and 73 DEGs, 55 and 277 5'-UTR, 18 and 152 3-UTR', 33 and 117 predicted RNAs (with 14 and 32 not-antisense RNAs and 19 and 85 antisense RNAs) in 1-R vs. 1-S and 2-R vs. 2-S. RNome studies on *Ab* ACICU RefGen evidenced 86 and 50 DEGs, 67 and 221 5'-UTR, 16 and 124 3-UTR', 33 and 102 predicted RNAs (with 12 and 22 not-antisense RNAs and 21 and 80 antisense RNAs) in 1-R vs. 1-S and 2-R vs. 2-S (S-Table_13A).

From the Short-Insert (SI) Libraries 2,353,045 - 2,041,858 - 1,804,167 - 1,819,349 total reads were produced in 1-S, 1-R, 2-S and 2-R, respectively, with 57, 56, 53, and 56% mapped reads on *Ab* ATCC 17978 and 59, 58, 54, and 57% on *Ab* ACICU. RNomes annotated on *Ab* ATCC 17978 RefGen revealed 77 and 28 DEGs, 211 and 94 5'-UTR, 94 and 48 3-UTR', 2909 and 1905 predicted RNAs (with 1497 and 1165 not-antisense RNAs and 1412 and 740 antisense RNAs) in 1-R vs. 1-S and 2-R vs. 2-S. An identical RNome analysis on *Ab* ACICU revealed 77 and 154 DEGs, 92 and 30 5'-UTR, 37 and 18 3-UTR', 388 and 245 predicted RNAs (with 151 and 107 not-antisense RNAs as well as 237 and 138 antisense RNAs) in 1-R vs. 1-S and 2-R vs. 2-S (S-Table_13B).

Integrating data obtained with the TS- and SI-library RNA-seq data, DAVID enrichment analysis of the COL-R vs. COL-S pairwise DEGs, obtained from the first filtering, evidenced a high variable strain-dependent response to the COL-R onset under COL-pressure.

Specifically, in 1 pair, the enrichment analysis of the pairwise DEGs showed 5 affected KEGG pathways (*p*-value ≤ 0.01) emerging within the count of the over-expressed DEGs mapped on *A. baumannii* ACICU RefGen, i.e. Butanoate metabolism (4 genes: ACICU_01343, ACICU_01342, ACICU_01735, ACICU_01767); Glycolysis/Gluconeogenesis (3 genes: ACICU_02656, ACICU_01735, ACICU_01737); Benzoate degradation via CoA ligation (3genes: ACICU_01343, ACICU_01342, ACICU_01767); Pyruvate metabolism (3 genes: ACICU_01735, ACICU_01737, ACICU_01767); Valine, leucine and isoleucine degradation (3 genes: ACICU_01408, ACICU_01342, ACICU_01767). Similarly, the enrichment analysis of the over-expressed DEGs annotated on *A. baumannii* ATCC 17978 showed 3 affected KEGG pathways including the Butanoate metabolism (5 genes: A1S_1729, A1S_1699, A1S_1341, A1S_2102, A1S_1705), Tryptophan metabolism (4 genes: A1S_1729, A1S_1341, A1S_2102, A1S_2450), Lysine degradation (3 genes: A1S_1729, A1S_1341, A1S_2102), Fatty acid metabolism, Limonene and pinene degradation (3 genes: A1S_1344, A1S_1341, A1S_2102), Benzoate degradation via CoA ligation (3 genes: A1S_1729, A1S_1344, A1S_1341). No KEGG pathways or GO-Biological processes were evidenced by the enrichment of the under-expressed DEGs in both annotations.

In 2 pair, DAVID enrichment analysis detected no affected KEGG pathways among the over-expressed DEGs on *Ab* ACICU annotation, on the contrary 23 biological process records (*p*-value ≤ 0.01) (A1S_1746, A1S_1813, A1S_0094, A1S_1529, A1S_0548, A1S_3247, A1S_1471, A1S_1330, A1S_2036, A1S_2042, A1S_1855, A1S_2751, A1S_1320, A1S_2750 genes) mainly attributable to genes involved in the regulation of transcription and gene expression were detected among the over-expressed DEGs assembled on *Ab* ATCC 17978. No KEGG pathways or Biological processes were evidenced by the DAVID enrichment of the under-expressed DEGs on both annotations.

On the integrated TS and SI library RNA-seq data and on *Ab* ATCC 17978 and ACICU RefGen annotations, the second comparative filtering analysis of the data sorting for the statistically significant differentially expressed genes with the same expression profile in both COL-R *Ab* strains showed 7 over-expressed protein-coding genes (*q*-value ≤ 0.01) for: (i) the PgaB outer membrane lipoprotein catalyzing the N-deacetylation of poly-beta-1,6-N-acetyl-D-glucosamine (PGA), an adhesin polysaccharide that promotes the PGA export associated with biofilm formation (A1S_0938), (Biological Process GO:0005976) (TIGR03938); (ii) the membrane non-ribosomal peptide synthetase module (A1S_2651), (COG:318) (GO:0008152), binding the phosphopantetheine (GO:0031177); (iii) the Lipid A phosphoethanolamine transferase PmrC involved in the phosphoethanolamine incorporation into lipidA (A1S_2752 and its homologous in ACICU_03004) (GO:0008152); (iv) the diacylglycerol kinase (ACICU_02907) for the lipid recycling in the membrane derived oligosaccharides cycle; (v) three hypothetical proteins (A1S_2027, A1S_2230 and its homologous ACICU_02436, and ACICU_01518) (Table 4).

**Table 4:** Transcriptomic traits characterizing COL-R *A. baumannii*.

***Ab* ATCC 17978Locus Tag^a^**	***Ab* ACICU ** ** Locus Tag^a^**	**Library^b^**	**Description**	**I-Tasser ** ***Ab* Initio Modeling Prediction**	**COG^c^**	**GO_numb.^d^**	**Expression of RNA-seq data annotated on** ***Ab*** **ATCC 17978** ^****a****^ **(*****q-value*** **≤0,01)**				**Expression of RNA-seq data annotated on** ***Ab*** **ACICU**^****a****^ **(*****q-value*** **≤** **0.01)**				**Function**	**Associated Phenotype**
							**1-R**	**1-S**	**2-R**	**2-S**	**1-R**	**1-S**	**2-R**	**2-S**		
A1S_0938	–	SI	Poly-beta-1,6-N-acetyl-D-glucosamine N-deacetylase PgaB	–	G	GO:0005976	81	20	392	40	–	–	–	–	Biofilm matrix production regulation	Biofilm producer
A1S_2651	–	SI	Membrane Non-ribosomal peptide synthetase	–	I, Q	GO:0008152	56	15	559	27	–	–	–	–	Siderophore production	Protection from ROS protection of (oxidative stress)
–	ACICU_02907	SI	Diacylglycerol kinase	–	M	GO:0008654	–	–	–	–	179	39	741	67	Recycle the diacylglycerol generated as a by-product of membrane-derived oligosaccharide biosynthesis	Integrity of cell membrane putatively aiding Colistin resistance
A1S_2752	ACICU_03004	SI	Lipid A phosphoethanol aminotransferase PmrC	–	R	GO:0008152	159	35	791	43	69	7	396	11	LPS modification	Colistin resistance
–	ACICU_01518	TS	Hypotetical protein	–	–	–	–	–	–	–	1042	253	3417	36	Membrane protein Periplasmatics	-
A1S_2027	-	TS	Hypotetical protein	RNA-binding motif	–	–	135	8	97	0	–	–	–	–	DNA damage response	Acquisition of mutational antibiotic resistance
A1S_2230	ACICU_02436	TS	Hypotetical protein	Photoreaction center of the photosynthesis	S	–	4551	0	949	11	3546	0	731	8	Light response	Motility, biofilm, MIN and TGC susceptibility

a Ab, Acinetobacter baumannii;

b SI, Short-Insert library; TS, Tru-Seq library;

c G, carbohydrate metabolism and transport; I, lipid metabolism; Q, secondary structure; R, general functional prediction only; S, unknown function;

d *GO numbers refer only to the biological process*.

### Validation of RNA-Seq Data

**Figure 4:**
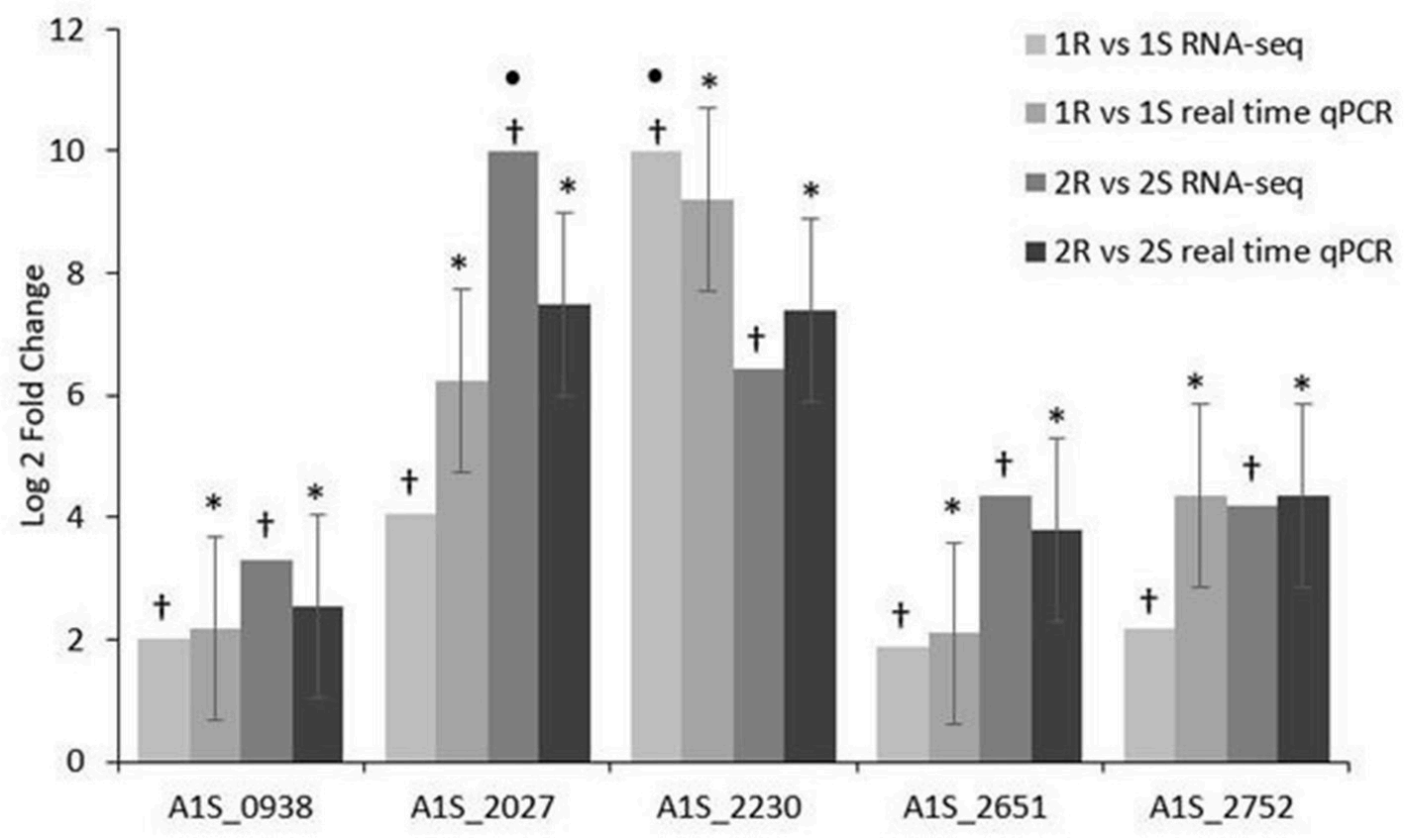
Real time qPCR validation of RNA-seq expression data on COL-R characterizing transcripts. • a value of 10-Fold Changes (FC) was indicated for incalculable FC due to the presence of a 0 value in one of the strains. *p-value ≤ 0.05 Statistical expression analyses were performed using the relative expression software tool 2009 (REST2009). †*q*-value ≤ 0.01 according to the Rockhopper guidelines.

The validation of the RNA-seq data performed by real time qRT-PCR assays confirmed that A1S_0938 Poly-beta-1,6-N-acetyl-D-glucosamine N-deacetylase *pgaB*, A1S_2027 hypothetical protein, A1S_2230/ACICU_02436 hypothetical protein, A1S_2651 membrane non-ribosomal peptide synthetase, and A1S_2752/ACICU_03004 Lipid A phosphoethanol aminotransferase *pmrC* had a statistically significant increased expression (*p*-value ≤ 0.05) in both COL-R *Ab* strains compared with their COL-S parents (Figure 4).

### I-Tasser *ab initio* Structure Modeling

I-Tasser *ab initio* structure modeling was used to computationally resolve the structure and function of the A1S_2027, A1S_2230 and ACICU_01518 hypothetical proteins.

Regarding the A1S_2027 hypothetical protein, the CD-BLAST of the A1S_2027 protein returned a homology with the KOW superfamily (cl00354). The KOW domain is known as an RNA-binding motif that is shared, so far, among some families of ribosomal proteins and the essential bacterial transcriptional elongation factor NusG. A1S_2027 contains a predicted alpha-helical coiled-coil and has a predicted cytoplasmatic localization. On the contrary, although the I-TASSER COACH Predicted function found, with a low C-score (0.03), the PDB-hit 1fosG of the human heterodimeric bZIP transcription factor c-Fos-c-Jun bound to DNA involved in transcription, the overview of generated predictions did not clarify the structure and biological function of the A1S_2027 protein. For the A1S_2230 hypothetical protein of unknown function, the CD-BLAST of the A1S_2230/ACICU_02436 protein found homology with the DUF2171 domain (pfam09939) (COG3798). This domain was found in hypothetical prokaryotic conserved proteins with no known function. The I-TASSER prediction of the threading templates, Gene Ontology (GO) and consensus prediction of GO terms of the A1S_2230 protein predicted the closest structural similarity with PDB hit 1e6dH (TM-score 0.730), GO-term 1prcH (0.26 C-score^GO^), consensus prediction of the GO term biological process (GO:0019684) and the cellular component (GO:0030077) with a 0.57 GO-Score, matching a plasma membrane protein of a Photoreaction Center of the photosynthesis characterized in *Rhodobacter sphaeroides* (Table 4).

Concerning ACICU_01518, unfortunately I-TASSER predictions did not clarify the structure and biological function of the ACICU_01518 protein.

### Transcripts of the Known Genes Associated With Colistin Resistance

**Figure 5:**
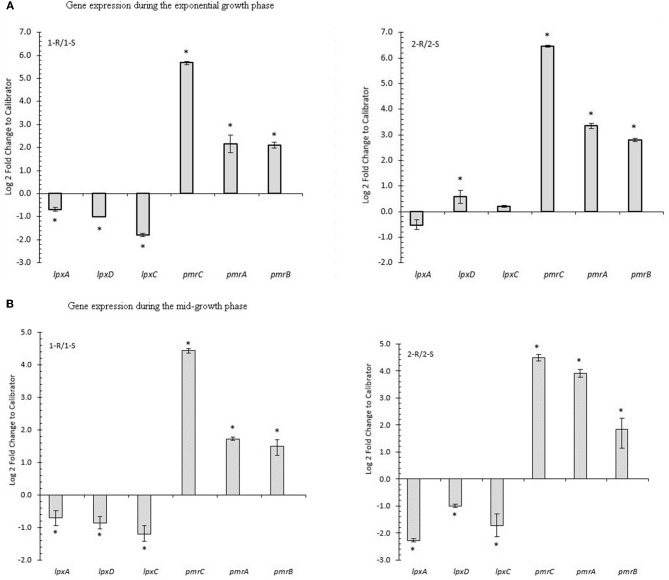
Gene expression of COL-R related genes in *A. baumannii*. **(A)** Gene expression during the exponential growth phase. **(B)** Gene expression during the mid-growth phase. ^*^p-value ≤ 0.05 Statistical expression analyses were performed using the relative expression software tool 2009 (REST2009).

*pmrCAB* transcript analysis showed a statistically significant over-expression of the *pmr*-operon with a notable increase in *pmrC*, both at the exponential and mid-log growth phases, comparing COL-R isolates with their COL-S counterparts. Conversely, a statistically significant *lpxACD* under-expression was observed during the mid-log phase for all COL-R strains and only in 1-R isolate during the exponential-growth phase (Figures 5A,B).

### Cell-Envelope Charge and LPS/SP Quantification

**Figure 6:**
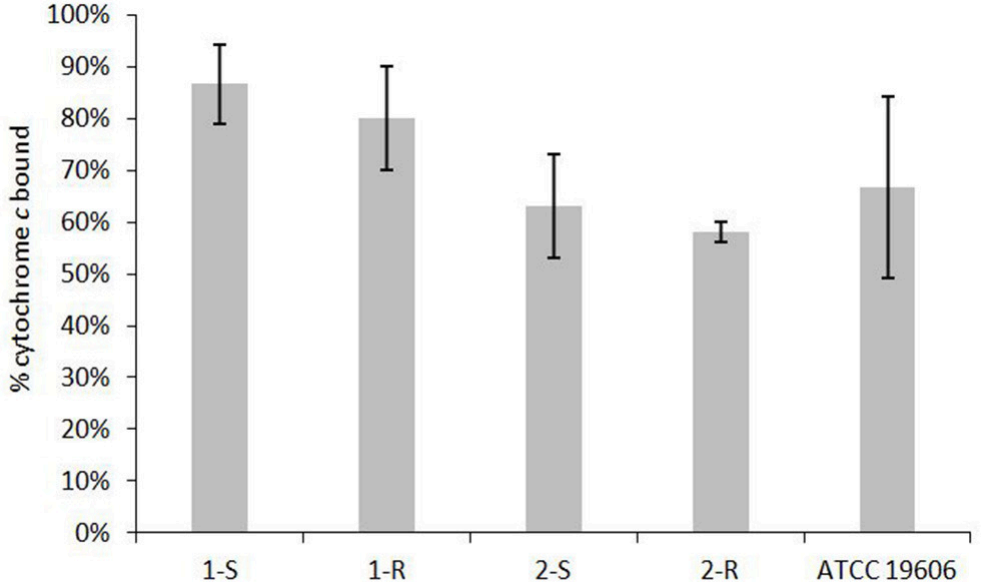
Relative positive surface charge by cytochrome *c* binding.

Determination of the net charge associated with the cell envelope showed a positive charge increase in the COL-R strains compared with their susceptible parent strain (Figure 6). A reduction in surface polysaccharide (SP) amount was displayed in 1-R, and less conspicuously in 2-R, whilst a LPS slight reduction occurred in both COL-R strains, although more evident in 2-R. However, these differences were not statistically significant (Table 5).

**Table 5:** Surface attached polysaccharide (indirect) and LPS (direct) quantification.

**Strain**	**SP amount ± SD (μg/mL)**	**LPS amount ± SD (μg/mL)**
1-S	144.84 ± 43.69	6298.40 ± 1267.04
1-R	71.770 ± 31.86	6002.95 ± 1178.74
2-S	178.38 ± 65.24	6003.80 ± 3717.05
2-R	124.54 ± 58.79	5184.77 ± 2526.66
COL-S *A. baumannii* ATCC 19606	170.69 ± 51.67	5843.86 ± 1102.01

## Discussion

*A. baumannii* is representative of the current public health crisis caused by the growing burden of infections associated with organisms that are resistant to most, if not all, antimicrobial agents. This species has proven to be particularly challenging because of its capacity to: (a) persist in hospital environments, (b) become resistant to antimicrobials and disinfectants, and (c) acquire new resistance determinants.

Two main colistin resistance mechanisms have been described in *Ab*. The first category consists of mutations in the PmrAB two-component regulatory system resulting in lipid A modification (Adams et al., 2009; Beceiro et al., 2011). The second category of resistance mechanisms evoke mutations or disruptions in genes encoding lipid A biosynthesis such as *lpxA, lpxC*, and *lpxD*, conferring the complete loss of LPS (Moffatt et al., 2010, 2011). In response to antibiotic pressure and its withdrawal, the *pmr*-locus can also go through mutational pathways determining, firstly, colistin resistance and, then, its loss together with a reduced probability to reacquire colistin resistance for the acquisition of compensatory inactivating pmr-mutations (Snitkin et al., 2013). Even though Pmr-locus mutations may not confer reduction in fitness, growth alteration, and virulence, an increase in growth retardation, reduced virulence and lower invasiveness in COL-R *Ab* isolates were also described (Andersson and Hughes, 2010; López-Rojas et al., 2013; Beceiro et al., 2014; Pournaras et al., 2014; Durante-Mangoni et al., 2015). Furthermore, adaptations to colistin treatment can occur following significant changes in physiological processes (Jawad et al., 1998; Dorsey et al., 2004; López-Rojas et al., 2011).

Recently, genome analysis at the population-level on MDR *Ab*, revealed new insights into their transmission and evolutionary dynamics during long-term infection demonstrating that the over-represented mutations acquired during infection were in transcriptional regulators, notably *pmrAB* and *adeRS*, mediating the resistance to colistin and tigecycline as well as transporters, surface structures, and iron acquisition genes (Wright et al., 2016).

Our analysis allowed a multi-level discovery of the genomics and transcriptomics COL-R *Ab* signatures.

The four *Ab* strains analyzed in this study were XDR isolates showing the same genomic macrorestriction profile A, belonged to the same Oxford University ST-281 and to Global Clone II, and with a very similar acquired resistome. This resemblance underline a genetic high similarity due to closely related genomes and phylogeny of our sample reflecting a transmission of specific phylogenetic strain clusters, strongly related also to *Ab* ACICU and *Ab* ATCC 17978 reference strains. This observation was further supported by the strong phylogenetic genomic correlation found between the two COL-S strains, sharing only 23 cSNP differences, whilst a high genomic diversity was recovered between the two COL-R *Ab* strains (81 cSNP differences) indicating that the acquisition of colistin resistance gain a high rate of genomic diversification respect to the COL-S parent strains.

In addition, all these considerations suggest that colistin resistance emerges in clinical settings from colistin susceptible strains going through the acquisition of new traits in agreement with recent observations (Mustapha et al., 2018) and our findings.

The whole genome sequencing and its SNP calling detected, in fact, common gSNPs in both COL-R strains vs. susceptible ones mapped on *Ab* ATCC 17978 and *Ab* ACICU. COL-R *Ab* accumulated several different nsSNPs, also associated to synonymous ones, in specific loci or distinct genes. Among these, we would like to highlight those affected proteins involved in COL-R mechanisms, i.e. A1S_2024/ACICU_01043 glutamate-5-kinase and A1S_2928 hypothetical protein previously associated with the intrinsic colistin resistance involved in resistance mechanisms (Olaitan et al., 2014), A1S_2928 related to the inducible colistin tolerance in response to physiologic concentrations of monovalent cations, that mediate osmotolerance-modulating pathways involved in compatible solute, cell envelope biosynthesis and protein folding (Hood et al., 2010), with a conserved domain similar to the stress-responsive protein Ish1 in yeast of the ACICU_02735, speculatively, indicating an involvement of adaptive mechanism in response to stress as colistin selective pressure. As regards the SNPs falling in genes non-directly related to COL-R, such as FilD and BenP porins along with A1S_2443 and ACICU_02910 surface adhesion proteins, we can speculate that these SNPs could affect different properties conditioning indirectly adaptation mechanisms of resistant strains through unknown pathways connecting drug-resistance and the accumulation of genetic mutations.

Conversely, synonymous SNPs that do not change the amino acid sequence in protein-encoding genes, because of genetic code redundancy, could impact translation efficiency and the amount of produced protein (Chaney and Clark, 2015), whilst SNPs that occur in non-coding regions are likely able to modify transcription factor binding or non-coding RNA sequences, this is understandable especially when located in promoter regions (Chaney and Clark, 2015).

To better investigate the transcriptomic profiling of COL-R vs. COL-S *Ab* strain pairs, we used two different libraries (single-end and paired-end with insert reads of different size) in the analysis of biological replicates, in order to minimize the biases due to the complexity of the RNA-seq procedures and algorithms for the processing of RNA-seq “big data”.

Comparative transcriptomic pairwise analysis highlighted a strain-dependent response to the colistin resistance onset highly variable among COL-R isolates, on the contrary the filtering of DEGs sorting for the COL-R common traits showed transcriptional changes consisting of seven common over-expressed genes in both COL-R *Ab* strains, representing the mRNA signatures of our COL-R *Ab*. In detail, we recorded an increased *pgaB* mRNA, for an outer membrane lipoprotein responsible for the N-deacetylation of PGA - a surface adhesin polysaccharide - in agreement with findings by other authors (Eijkelkamp et al., 2014). The *pgaB* over-expression increased the primary amine content (glucosamine of PGA via PGA deacetylation) promoting their export through a PgaA porin (A1S_0938) associated with biofilm formation (Choi et al., 2009). The consequent positively charged PNAG can indirectly inhibit the activity of cationic colistin on LPS. In addition, polysaccharide deacetylase was related to cell-wall modification (Ramazzina et al., 2008) and it can modulate the uptake of antibiotics, including colistin. Hence, a glycosyltransferase (PNAG deacetylase) could be part of an interaction network affecting the outer membrane and cell-wall integrity, giving a further contribution to the colistin resistance in *Ab* (Park et al., 2015).

Regarding the over-expressed A1S_2027 mRNA, this signature suggests that COL-R *Ab* isolates are distinguished by an induction of the transcriptional response to DNA damage related to the acquisition of antibiotic resistance and, thus, to their Multi-Drug-Resistance (Hare et al., 2014). Although it was not possible to determine the A1S_2027 precise structure and functionality, we found that it was previously described as a hypothetical phage protein involved in the complex network of *A. baumannii* and *A. baylyi* DNA damage response regulated by *recA*. Furthermore, Norton et al. (2013) demonstrated that the DNA damage response increases mutagenesis and it is one of the mechanisms used by *Ab* to acquire antibiotic resistance under clinically relevant DNA-damaging conditions (Hua et al., 2017).

The over-expressed A1S_2230/ACICU_02436 mRNA, according to the results of a proteome study (Mussi et al., 2010), may be a signature related to the light response of COL-R *Ab*, also related to their TGC susceptibility. For this mRNA, the computational predictions found similarity with a protein of a photoreaction center of the photosynthesis characterized in *Rhodobacter sphaeroides*. Surprisingly, we found that in the genome of *Ab* ATCC 17978, the A1S_2230 tag is located near A1S_2225, previously described as encoding the BlsA (blue-light-sensing A) *A. baumannii* photoreceptor protein. Mussi et al. (2010) demonstrated that *A. baumannii* senses and responds to blue light. These bacterial responses depended on the expression of the *Ab* ATCC 17978 A1S_2225 gene named blue-light-sensing A (*blsA*). The light modulated motility and biofilm formation (Ramírez et al., 2015a), more precisely motility and formation of biofilms and pellicles, were observed only when bacterial cells were incubated in darkness. Furthermore, the same authors demonstrated that susceptibility to minocycline and tigecycline, two antibiotics used for the treatment of MDR *A. baumannii* infections, was modulated by light (Ramírez et al., 2015b). Beyond susceptibility to antibiotics, this response to light might enhance the ability of the bacterium to persist until conditions are more favorable for growth or accumulation of additional resistance determinants, directly impacting the microorganism's ability to persist in the environment.

Regarding the over-expressed non-ribosomal peptide synthetase module A1S_2651, in agreement to previous findings (Dwyer et al., 2009; Adler et al., 2012; Wright et al., 2017), this typical COL-R trait could be related to a high biosynthesis of a siderophore for iron coordination implicated in protection from reactive oxygen species (ROS), by chelating free iron, and in the protection of oxidative stress.

As regards the over-expression of ACICU_02907 encoding a diacylglycerol kinase (DAGK), a small integral membrane protein (Smith et al., 1994), this transcriptomic trait evidenced that COL-R *Ab* have an increased recycle of diacylglycerol (DAG), by-product of membrane-derived oligosaccharide biosynthesis (Walsh and Bell, 1992), in phospholipids in the cell. This metabolic adaptation was previously related to the integrity of the cell membrane and aided colistin resistance although with an unknown mechanism. Furthermore, if the amount of DAG in the lipid bilayer becomes too high, cells lose their ability to proliferate in mildly stressful environments (Badola and Sanders, 1997). Furthermore, the over-expressed ACICU_01518 hypothetical protein of unknown function was previously found with an increased expression in COL-R laboratory-derived strains under *in vitro* colistin selection (Park et al., 2015).

The up-regulation of lipid A phosphoethanolamine transferase PmrC - likely in association with *pmrB* SNPs-, evidenced by RNA-seq as well as in exponential and mid-log growth-phase real time qPCR data, strongly supports its key role in COL-resistance in *Ab* due to a lipid A phosphoethanolamine modification that determines a change in the cell surface charge responsible for a repulsion between positive colistin and the more positively charged phosphoethanolamine-modified LPS. These results, in agreement with previous findings (Arroyo et al., 2011; Lim et al., 2015; Choi et al., 2017), demonstrated that specific alterations in *pmr*CAB, such as the PmrB L208F or R263H in histidine kinase domain along with the firstly found L340 in the PmrC sulfatase domain, are responsible for an increase of colistin MIC and associated with *pmrBC* over-expression and polymyxin resistance in our *A. baumannii*.

Regarding the colistin resistance mechanisms mediated by lpx-cluster, previously described by other authors in “*in vitro*-derived” mutants (Moffatt et al., 2010), for the first time the involvement of *lpxC* and *lpxD* nsSNPs was demonstrated in a clinical COL-R Ab. In detail, we found a S171Y AA substitution in the phosphoethanolamine transferase N-terminal LpxC domain and a S292 in the LpxD UDP-3-O-(3-hydroxymyristoyl) Glucosamine N-acyltransferase domain generating a premature stop codon. These results suggested either that a LpxD premature stop codon at the 292 AA did not entirely affect LpxD functionality and LPS biosynthesis, or that a functional LpxD could be not required for LPS biosynthesis in our clinical COL-R *Ab*. Anyhow, this LpxD condition, together with the under-expression in the S171Y modified-LpxC transcript and *lpxA*, could explain at least theoretically the decreased LPS amount displayed in this strain. In addition, more in general, our data on *lpxACD* support the relation between *lpx*-nsSNP occurrence and under-expression with the colistin-resistance onset in *Ab* strains.

In conclusion, genomic and transcriptomic signatures of COL-R vs. COL-S *Ab* strains revealed main and secondary accessory traits, reflecting the complexity of these microorganisms in the continuous balance between their antimicrobial resistance and virulence that interplay for the survival in the host under antimicrobial pressure.

Our study defined nsSNPs hotspots and transcriptomic traits correlating to the colistin resistance onset and demonstrated the involvement of many adaptation features occurring in loci indirectly related to the antibiotic resistance. It seems being clear that becoming COL-R is a complex event in which LPS as a target is involved, but it is not the exclusive one. Indeed, new signatures and indirect pathways are also implicated.

## Author Contributions

VC and SS conceived and designed the study. VC, SStr, FL, and GG performed the Genomics, Transcriptomics, real time qPCR and bioinformatics. MM and CC performed the MICs and Molecular Typing. GP and AF contributed to the bioinformatics analysis. All authors analyzed the data and contributed to the manuscript.

### Conflict of Interest Statement

The authors declare that the research was conducted in the absence of any commercial or financial relationships that could be construed as a potential conflict of interest.
