# Supplementary material for: Colistin Resistant A. baumannii: Genomic and Transcriptomic Traits Acquired Under Colistin Therapy
Source: Front Microbiol. 2019 Jan 7;9:3195. doi: 10.3389/fmicb.2018.03195 (PMC6330354; doi:10.3389/fmicb.2018.03195)
Supplement: Supplementary file 1 [file Table_1.DOCX]

**S-Table_1. Total number of paired end (PE) and mate pair (MP) reads with the estimated coverage**

| **Sample** | **Filtered PE (M)** | **Filtered MP (M)** | **E. Coverage** |
| --- | --- | --- | --- |
| **1-S** | 4,45M(*2) | 1,31M(*2) | 249,88X |
| **1-R** | 0,77M(*2) | 1,47M(*2) | 121,63X |
| **2-S** | 2,03M(*2) | 1,14M(*2) | 148,11X |
| **2-R** | 3,24M(*2) | 0,93M(*2) | 180,89X |
