# Supplementary material for: Colistin Resistant A. baumannii: Genomic and Transcriptomic Traits Acquired Under Colistin Therapy
Source: Front Microbiol. 2019 Jan 7;9:3195. doi: 10.3389/fmicb.2018.03195 (PMC6330354; doi:10.3389/fmicb.2018.03195)
Supplement: Supplementary file 2 [file Table_2.DOCX]

**S-Table_2. Table of Assembly Statistics**

| **Sample** | **Contigs**  **(≥ 0bp)** | **Contigs**  **(≥ 500bp)** | **Contigs**  **(≥1000bp)** | **N50** |
| --- | --- | --- | --- | --- |
| **1-S** | 188 | 59 | 50 | 427394 |
| **1-R** | 173 | 75 | 57 | 427117 |
| **2-S** | 151 | 68 | 55 | 359072 |
| **2-R** | 190 | 55 | 46 | 432770 |
