# Supplementary material for: Colistin Resistant A. baumannii: Genomic and Transcriptomic Traits Acquired Under Colistin Therapy
Source: Front Microbiol. 2019 Jan 7;9:3195. doi: 10.3389/fmicb.2018.03195 (PMC6330354; doi:10.3389/fmicb.2018.03195)
Supplement: Supplementary file 3 [file Table_3.DOCX]

**S-Table_3. Sample coverage estimation for sample’s reference genomes**

| **Sample** | **Mapped Reads**  **(M)** | **Perc. Mapped**  **(%)** | **Genome Size**  **(Mb)** | **Coverage**  **(X)** |
| --- | --- | --- | --- | --- |
| **1-S** | 3,85 | 87.94 | 3,98 | 116,3 |
| **1-R** | 0,68 | 87.95 | 3,98 | 20,45 |
| **2-S** | 1,74 | 87.10 | 3,98 | 52,52 |
| **2-R** | 2,79 | 87.76 | 3,98 | 84,06 |
