# Supplementary material for: Colistin Resistant A. baumannii: Genomic and Transcriptomic Traits Acquired Under Colistin Therapy
Source: Front Microbiol. 2019 Jan 7;9:3195. doi: 10.3389/fmicb.2018.03195 (PMC6330354; doi:10.3389/fmicb.2018.03195)
Supplement: Supplementary file 12 [file Table_12.DOCX]

**S-Table_12. Primer set used in real time qPCR**

| **Primer** | **Sequences** | **Fragment Size (bp)** |
| --- | --- | --- |
| ***lpx*A-up** | 5’ AACCACCTACAACCACATGAGAAT 3’ | 104 |
| ***lpx*A-dn** | 5’ ACCGCCATTATTGATCCATCTGC 3’ |  |
| ***lpx*C-up** | 5’ ACAACACCCGTATCATCTACACCA 3’ | 117 |
| ***lpx*C-dn** | 5’ ATGAAGTCAGTGAGGCACGAACT 3’ |  |
| ***lpx*D-up** | 5’ TGCTTTCTATGCCTGTTCAGC 3’ | 138 |
| ***lpx*D-dn** | 5’ CGCTTACATTGTTACCGCAGC 3’ |  |
| ***pmr*A-up** | 5’ CGTCAAAGAGCAGCAACACCAGTA 3’ | 130 |
| ***pmr*A-dn** | 5’ CGGGCAAGCAACTCATCAAACTCA 3’ |  |
| ***pmr*B-up** | 5’ CATTTGCTGGTTCCACCTGTTGAG 3’ | 123 |
| ***pmr*B-dn** | 5’ CCCTCTCTTGCTGACTGACCTGA 3’ |  |
| ***pmr*C-up** | 5’ TTGCCAAAGATGATGATCGCCCAC 3’ | 134 |
| ***pmr*C-dn** | 5’ AGCCCTGTATCGCATTCGTATCAC 3’ |  |
| ***rpo*B-up** | 5’ CAAGAGTCTAATGGCGGTGGTTCA 3’ | 115 |
| ***rpo*B-dn** | 5’ GCGATTGCTTCATCTGCTGGTTG 3’ |  |
| **A1S_0938-up** | 5’ ATGCTTCAACACTTACGGTCATT 3’ | 230 |
| **A1S_0938-dn** | 5’GAAAGACTGATAACCATCATCTACAGT3’ |  |
| **A1S_2027-up** | 5’ TTGCTCGTTCTAAGTACATTCAAG3’ | 214 |
| **A1S_2027-dn** | 5’ ATTAAGTCTTTATGCTGGTATCCAC 3’ |  |
| **A1S_2230-up** | 5' AAGTTTCTCTCTAAATGGGTATGC ’3’ | 191 |
| **A1S_2230-dn** | 5’ ATCGCTTGCCAGTTTTCTTTAACT 3’ |  |
| **A1S_2651-up** | 5’GAATGATTACTACAGATGAATGGTATG3’ | 256 |
| **A1S_2651-dn** | 5’ TAGACCTTGTCTTGCTCTTGAATAC 3’ |  |
| **A1S_2752-up** | 5’ AAGTTTCTCTCTAAATGGGTATGC 3’ | 211 |
| **A1S_2752-dn** | 5’ TTGGTAACCCGCACGTTTTG 3’ |  |
