# Supplementary material for: Colistin Resistant A. baumannii: Genomic and Transcriptomic Traits Acquired Under Colistin Therapy
Source: Front Microbiol. 2019 Jan 7;9:3195. doi: 10.3389/fmicb.2018.03195 (PMC6330354; doi:10.3389/fmicb.2018.03195)
Supplement: Supplementary file 13 [file Table_13.DOCX]

**S-Table_13. RNAomes**

A) Tru-seq Library Rockhopper Summary

| **Tru-seq Libraries**  **Rockhopper RNAome Summary** | **1-S** | **1-R** | **2-S** | **2-R** |
| --- | --- | --- | --- | --- |
| **Total Reads** | 1,307,792 | 1,175,327 | 1,173,332 | 1,270,020 |
| **Mapped reads**  **on *Ab* ATCC17978** | 96%  (1,249,622) | 96%  (1,128,614) | 72%  (847,884) | 93%  (1,185,605) |
| **Mapped reads**  **on *Ab* ACICU** | 97%  (1,264,432) | 97%  (1,139,137) | 76%  (894,626 | 95%  (1,209,661) |
|  |  |  |  |  |
|  | **1-S and 1-R 2-S and 2-R**  **on *Ab* ATCC17978** | | **1-S and 1-R 2-S and 2-R**  **on *Ab* ACICU** | |
| **5'-UTR** | 55 | 277 | 67 | 221 |
| **3'-UTR** | 18 | 152 | 16 | 124 |
| **Predicted RNA** | 33 | 117 | 33 | 102 |
| **not antisense RNAs** | 14 | 32 | 12 | 22 |
| **antisense RNAs** | 19 | 85 | 21 | 80 |
| **Differentially Expressed Genes** | 47 | 73 | 86 | 50 |
| **Gene-pairs predicted as likely operons** | 343 | 404 | 1290 | 1394 |
| **Multi-gene operon** | 286 | 325 | 669 | 685 |

B) Short-Insert Library Rockhopper Summary

| **SI Libraries**  **Rockhopper RNAome Summary** | **1-S** | **1-R** | **2-S** | **2-R** |
| --- | --- | --- | --- | --- |
| **Total Reads** | 2,353,045 | 2,041,858 | 1,804,167 | 1,819,349 |
| **Mapped reads**  **on *Ab***  **ATCC17978** | 57%  (1,339,126) | 56%  (1,139,927) | 53%  (961,806) | 56%  (1,021,138) |
| **Mapped reads**  **on *Ab* ACICU** | 59%  (1,378,651) | 58%  (1,183,018) | 54%  (978,770) | 57%  (1,038,587) |
|  |  |  |  |  |
|  | **1-S and 1-R 2-S and 2-R**  **on *Ab* ATCC17978** | | **1-S and 1-R 2-S and 2-R**  **on *Ab* ACICU** | |
| **5'-UTR** | 211 | 94 | 92 | 30 |
| **3'-UTR** | 94 | 48 | 37 | 18 |
| **Predicted RNA** | 2909 | 1905 | 388 | 245 |
| **not antisense RNAs** | 1497 | 1165 | 151 | 107 |
| **antisense RNAs** | 1412 | 740 | 237 | 138 |
| **Differentially Expressed Genes** | 77 | 28 | 77 | 154 |
| **Gene-pairs predicted as likely operons** | 330 | 333 | 1316 | 1282 |
| **Multi-gene operon** | 278 | 279 | 667 | 676 |
