# Supplementary figures and images for: Colistin Resistant A. baumannii: Genomic and Transcriptomic Traits Acquired Under Colistin Therapy
Source: Front Microbiol. 2019 Jan 7;9:3195. doi: 10.3389/fmicb.2018.03195 (PMC6330354; doi:10.3389/fmicb.2018.03195)

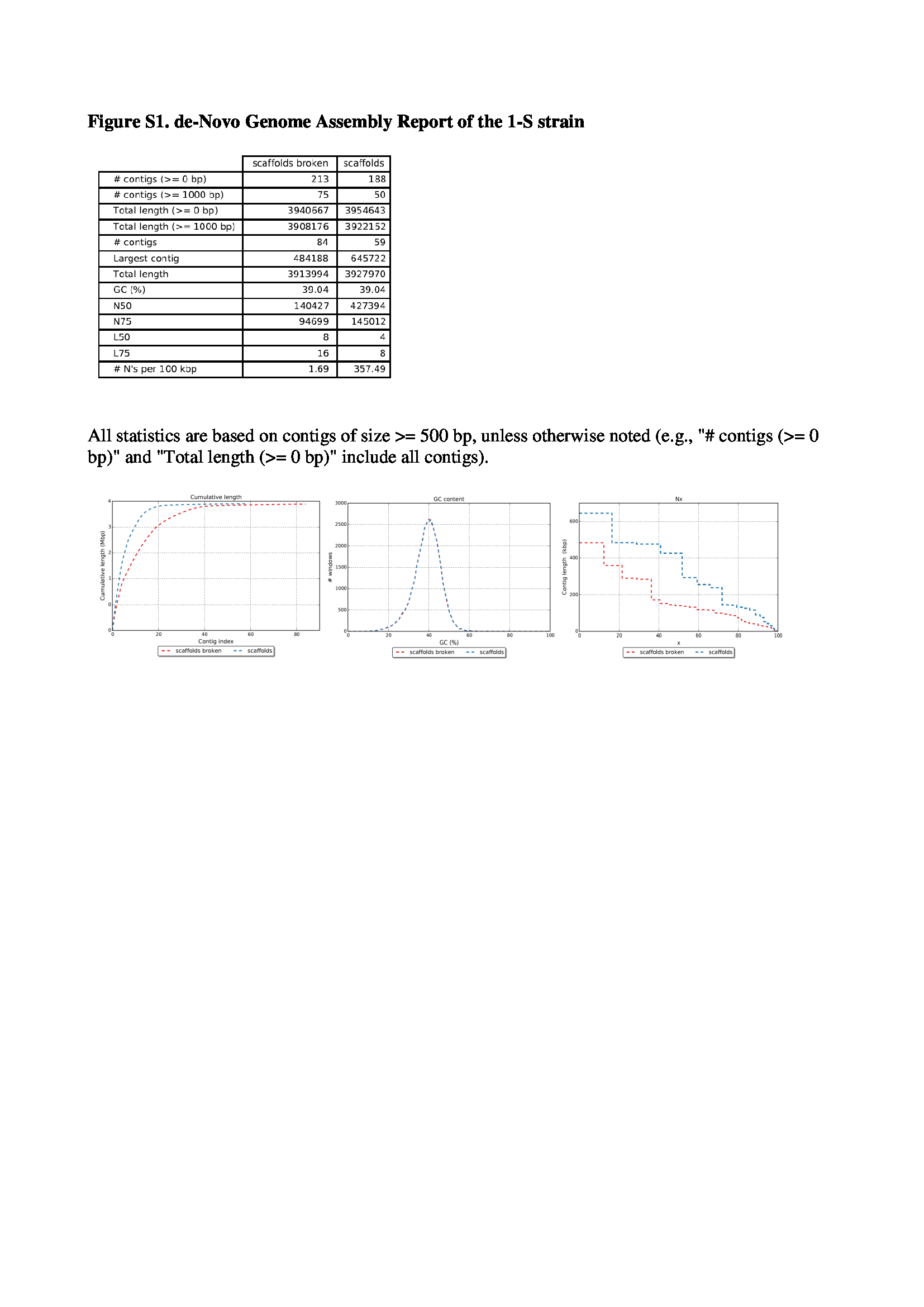

Supplement: Supplementary file 14 [file Image_1.JPEG]

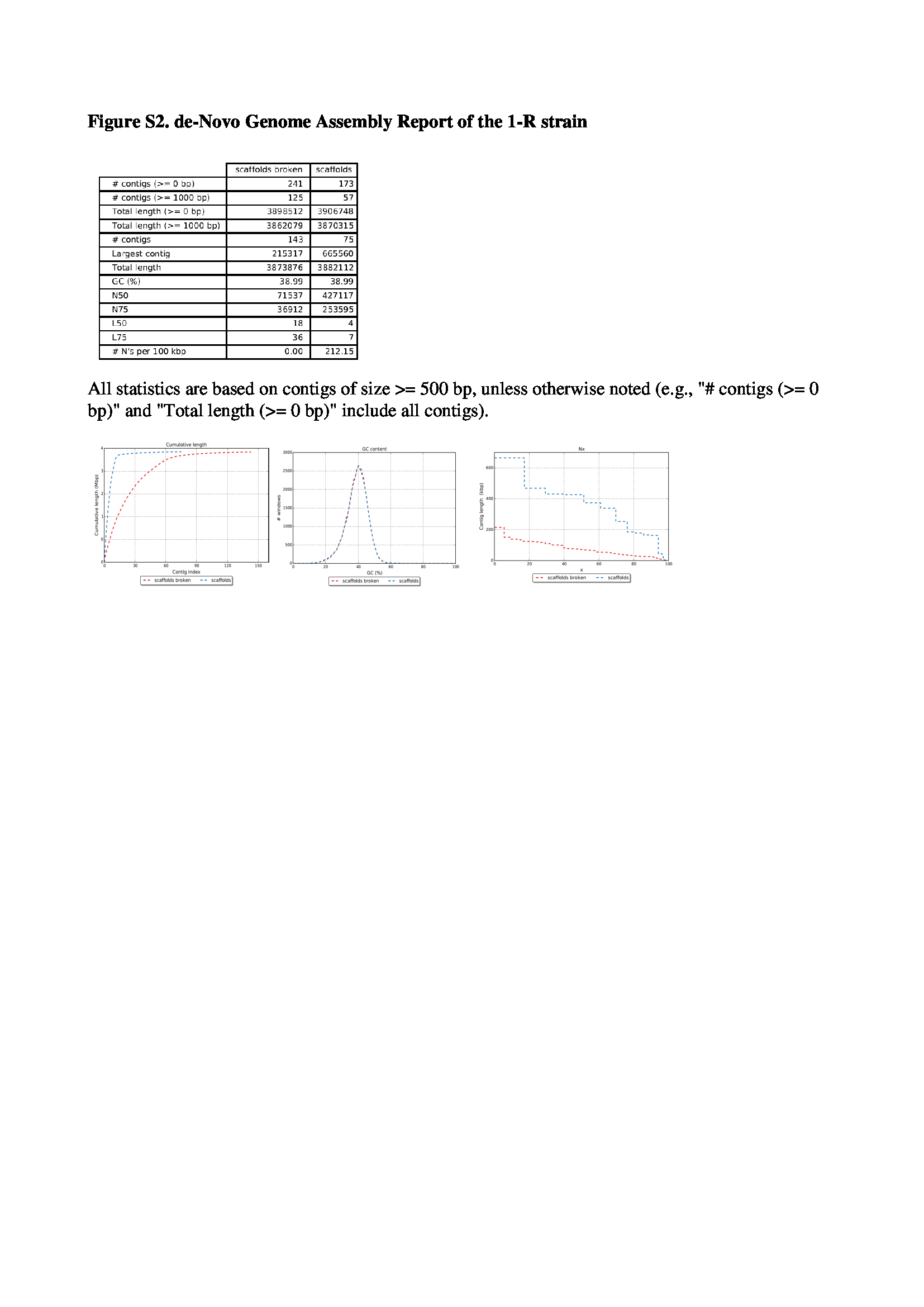

Supplement: Supplementary file 15 [file Image_2.JPEG]

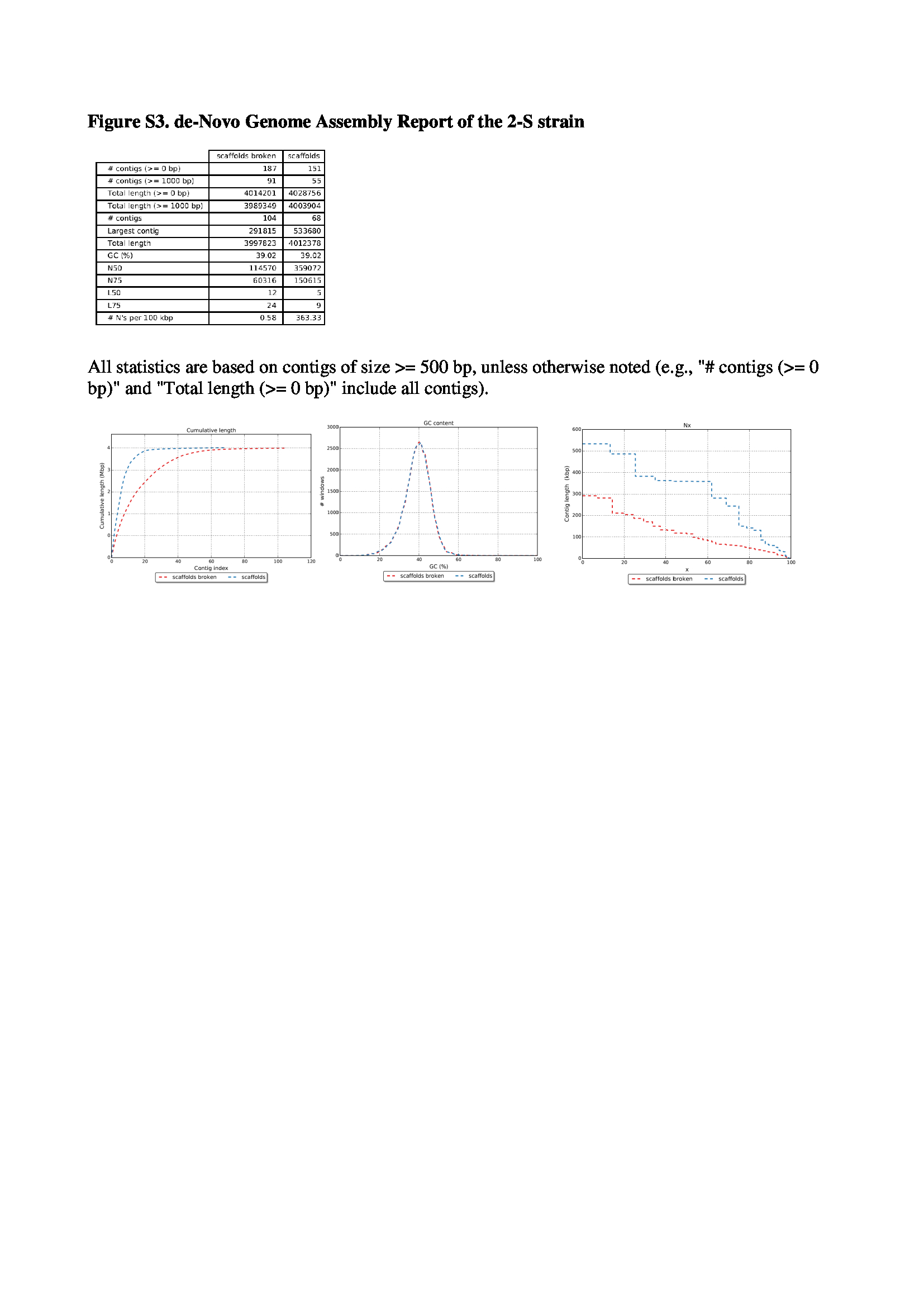

Supplement: Supplementary file 16 [file Image_3.JPEG]

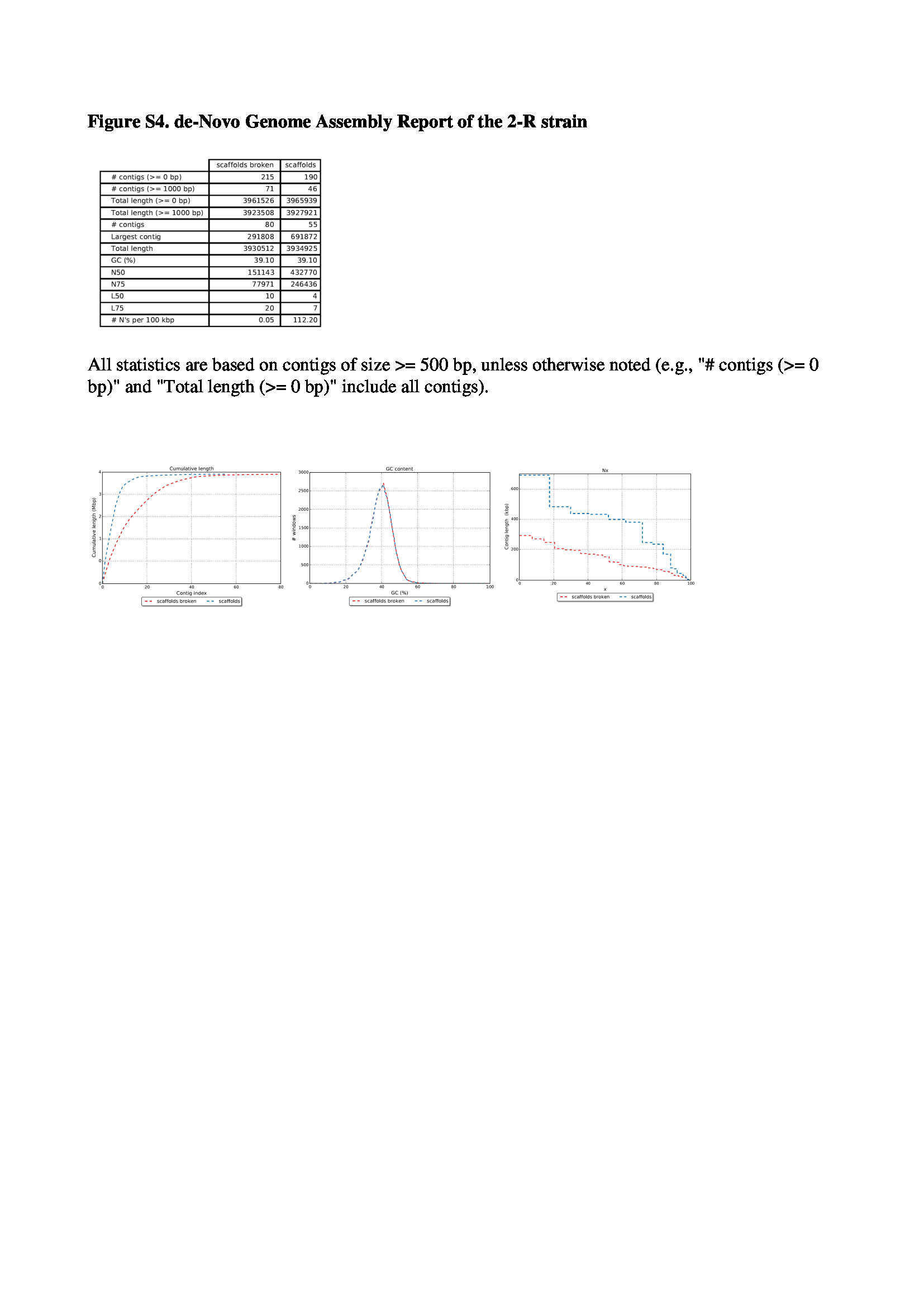

Supplement: Supplementary file 17 [file Image_4.JPEG]
